# Preoperative Microbiomes and Intestinal Barrier Function Can Differentiate Prodromal Alzheimer’s Disease From Normal Neurocognition in Elderly Patients Scheduled to Undergo Orthopedic Surgery

**DOI:** 10.3389/fcimb.2021.592842

**Published:** 2021-03-29

**Authors:** Mei Duan, Fangyan Liu, Huiqun Fu, Shibao Lu, Tianlong Wang

**Affiliations:** ^1^ Department of Anesthesiology, Xuanwu Hospital, Beijing, China; ^2^ Department of Orthopedics, Xuanwu Hospital, Beijing, China

**Keywords:** elderly patients, orthopedic surgery, preoperative period, prodromal Alzheimer’s disease, intestinal microbial dysbiosis, intestinal barrier dysfunction, systemic inflammation

## Abstract

**Objective:**

Emerging evidence links perturbations in the microbiome to neurodegeneration in amnestic mild cognitive impairment (aMCI) and Alzheimer’s disease (AD) and to surgical stress. In this study, we attempted to identify preoperative differences intestinal microbiota (IM) and barrier function between pAD [prodromal AD: Subjective cognitive decline (SCD) and aMCI] patients and normal neurocognition (NC) patients. Additionally, the potential associations between IM and barrier function, inflammation, and the clinical characteristics of pAD were evaluated.

**Design:**

Eighty elderly patients scheduled to undergo orthopedic surgery were consecutively enrolled and grouped as NC, SCD, and aMCI following neuropsychological assessment. IM was determined by 16S rRNA MiSeq sequencing, and PICRUSt was used to predict functional shifts in IM. Furthermore, we investigated the association between IM and plasma claudin-1, occludin, LPS, systemic inflammatory cytokines, neuropsychological assessment, and clinical characteristics.

**Results:**

There was a lower Chao1 index in the SCD group (P = 0.004) and differences in beta diversity among the three groups (PCA: P = 0.026, PCoA: P= 0.004). The relative abundance of *Bacteroidetes* was higher in the SCD group (P = 0.016, P = 0.008), and *Firmicutes* were more enriched in the aMCI group than in the SCD group (P= 0.026). At the family level, the total abundance of Gram-negative bacteria was higher in the SCD group than in the aMCI group (P = 0.047), and the *Christensenellaceae* family was detected at lower levels in the SCD and aMCI groups than in the NC group (P= 0.039). At the genus level, the eleven short-chain fatty acid (SCFA)-producing bacteria exhibited differences among the three groups. PICRUSt analysis showed that the pathways involved in SCFA catabolism, biosynthesis, and adherent junctions were reduced in SCD patients, and lipid synthesis proteins were reduced in pAD patients. Meanwhile, elevated plasma LPS and CRP were observed in SCD patients, and higher plasma occludin in aMCI patients. The IM was correlated with plasma claudin-1, LPS, inflammatory factors, neuropsychological assessment, and clinical characteristics.

**Conclusion:**

The intestines of SCD and aMCI patients preoperatively exhibited IM dysbiosis and barrier dysfunction, and elevated plasma LPS and CRP were observed in SCD patients.

## Introduction

AD typically progresses in three stages: the preclinical stage, the mild cognitive impairment (MCI) stage, and the dementia stage (Sperling et al., 2011). Emerging evidence links perturbations in the microbiome to neurodegeneration and Alzheimer’s disease (AD) (Tremlett et al., 2017; Vogt et al., 2017). In addition, intestinal microbial dysbiosis has been observed in patients with pAD, including those suffering from MCI and amnestic MCI (aMCI) due to AD (Li B. et al., 2019; Liu P. et al., 2019). Recently, it was observed that the composition and diversity of gut microbiota is altered in patients following surgery (Zhu et al., 2018). However, to the best of our knowledge, no reports have described the preoperative gut microbiota in SCD and aMCI patients prior to surgery.

Intestinal microbial dysbiosis in AD and pAD patients is characterized by an increase in proinflammatory bacteria and a decrease in anti-inflammatory bacteria (Cattaneo et al., 2016). Gram-negative bacteria, such as *Escherichia* and *Shigella*, are associated with brain amyloidosis in patients with neurocognitive impairment (Cattaneo et al., 2016). Gram-negative bacteria may induce intestinal barrier dysfunction (Wang et al., 2019), resulting in the translocation of Gram-negative bacteria-derived LPS either into the blood or directly into the brain. Bacterial LPS may induce systemic inflammation, neuroinflammation, and pathological processes that are associated with amyloidosis and impaired neurocognitive function (Brown, 2019). Furthermore, *Bacteroides fragilis* lipopolysaccharide, a GI tract microbiome-derived LPS, directly downregulates neuron-specific neurofilaments and pre- and postsynaptic proteins, thereby damaging neurons *in vitro* (Zhao et al., 2019). Decreased levels of Gram-negative bacteria may slow the progression of AD by inhibiting systemic inflammation, which may reduce the accumulation of amyloid beta aggregates and brain damage (Bonfili et al., 2017).

In addition, the levels of SCFA-producing bacteria were reduced in MCI and AD patients (Cattaneo et al., 2016; Tran et al., 2019). SCFAs are synthesized in the colon by SCFA-producing bacteria (Morrison and Preston, 2016) and function as a source of energy for intestinal cells (Clausen and Mortensen, 1995), which increases the expression of the tight junction proteins occludin and claudin-1 (Wang, 2012). Therefore, these bacteria can protect gut mucosal permeability and inhibit the translocation of LPS into the submucosae and blood (Wang, 2012). Decreases in the levels of SCFA-producing bacteria may exacerbate colonic epithelial permeability and LPS translocation into blood (Holota et al., 2019), thereby causing systemic inflammation, neuroinflammation, and neurodegeneration by entering the brain (Brown, 2019). Furthermore, SCFAs, particularly propionate and butyrate, can inhibit the inflammatory response both *in vitro* and *in vivo* (Nastasi et al., 2015). Decreased levels of SCFA-producing bacteria in MCI patients are associated with increases in the systemic proinflammatory cytokines IL-1β, NLRP3, and CXCL2 and brain amyloidosis (Nastasi et al., 2015). In contrast, butyrate-producing bacteria may restore the production of SCFAs in the gut, thereby preventing or ameliorating AD pathology (Nagpal et al., 2019). Furthermore, butyrate feeding can significantly improve memory function and AD markers in an AD mouse model (Govindarajan et al., 2011). The findings of these studies indicate that elevated levels of Gram-negative bacteria, decreased levels of SCFA-producing bacteria, intestinal barrier dysfunction, systemic inflammation, and neuroinflammation may all be correlated with cognitive impairment.

In this study, we investigated the preoperative differences in IM, intestinal barrier dysfunction, and low-grade inflammation between pAD patients and patients with normal cognition, and we investigated the association between the gut microbiota, as reflected by preoperative fecal samples, and intestinal linker proteins, plasma LPS, systemic inflammatory cytokines, neuropsychological assessment, and clinical characteristics.

## Materials and Methods

### Clinical Design

The research was designed as a case-control study and was approved by the Ethics Committee of XuanWu Hospital at Capital Medical University. All patients provided signed informed consent before sample collection. We chose 65~79-year-old patients with at least 9 years of education for orthopedic surgery. Sample size was calculated based on our preliminary result of the plasma endotoxin measurements. To achieve 80% power at an α level of 0.05 (two-tailed), at least 30 subjects were needed in each group. At first, we recruited 90 patients, including 36 patients of NC, 29 patients of SCD, 25 patients of aMCI. And 3 patients of SCD and 7 patients of aMCI dropped out the study. Finally, 36 patients of NC, 26 patients of SCD, and 18 patients of aMCI enrolled in this study. There were 22 patients with lumbar disc protrusion and 58 patients with knee arthritis in our study. Each participant underwent a complete medical history evaluation, neuropsychological assessment including the Montreal Cognitive Assessment-Basic (MoCA-B), fecal sample collection, and standard laboratory tests.

### Clinical Data Collection

Demographic data were collected 1 day before surgery. Vital signs (blood pressure, heart rate, electrocardiography, and pulse oxygen saturation) and blood gas (ABL800 FLEX; Radiometer Medical, Denmark) were measured for all patients before surgery.

A comprehensive neuropsychological test battery that was designed to examine four cognitive domains was administered to each participant by the same neurologist at Xuanwu Hospital: 1) Memory: AVLT-H (Auditory Verbal Learning Test-Hua Shan), which was adapted from the California Verbal Learning Test, presenting 12 words over five trials, and the scores on immediate recall, short-delay free recall (5 min), long-delay free recall (20 min), and long-delay recognition were derived from Guo et al. (2007), while the score on the five words-long-delay free recall was derived from the Montreal Cognitive Assessment-Basic (MoCA-B) (Hua Shan Hospital, Guo QiHao) (Chen et al., 2016); 2) Executive function was examined by the Trail Making Test B (STT-B) (Zhao et al., 2013a) and the Clock-Drawing Test (CDT-30) (Guo et al., 2008); 3) Language was examined by a Semantic Verbal Fluency Test (category, animals and fruits) (Zhao et al., 2013b) and 4) Visuoconstructive skill was examined by the CDT-30. In addition, the MoCA-B was performed to assess the condition of global cognition. The MoCA-B assesses nine cognitive domains (executive function, language, orientation, calculation, conceptual thinking, memory, visuoperception, attention, and concentration) and is freely available for clinical use (www.mocatest.org, visit the Basic section). Clinical Dementia Rating Scale (CDR) (Morris, 1993) was used for evaluating the severity of dementia [Dementia was diagnosed according to the Diagnostic and Statistical Manual of Mental Disorders, fourth edition (DSM-IV) (Chen et al., 2016) criteria for dementia. And CDR = 1]. The Activity of Daily Living (ADL) (He et al., 1990) was administered for the evaluation of social functioning. The Hachinski Ischemic Index (HIS) (Iliff et al., 1975) was used to differentiate between degenerative and vascular etiologies.

The neuropsychological standard ADNI 2 (Alzheimer’s disease neuroimaging initiative 2) was applied for the classification of aMCI, SCD, and NC (Baars et al., 2008; Jessen et al., 2014). NC was assigned when participants did not have SCD complaints and achieved a normal score [>1.5 standard deviations (SD) cutoff] in all four cognitive domains (memory, executive function, language, or visuoconstructive skill) and MoCA-B. Additionally, the CDR score was required to equal 0 in NC. The inclusion criteria of aMCI patients were as follows: (1) definite complaints of memory decline, preferably confirmed by an informant; (2) objective cognitive performances in single or multiple domains, including memory documented by neuropsychological test scores were below or equal to 1.5 SD of age- and education-adjusted norms; (3) a Clinical Dementia Rating (CDR) score of 0.5; (4) preservation of independence in activities of daily living; and (5) insufficient to meet the criteria for dementia based on DSM-IV-R (Diagnostic and Statistical Manual of Mental Disorders, 4th edition, revised). For SCD, there were the following requirements: (1) answer of “yes” to the question “Do you have problems in memory?”; (2) performance on standardized neuropsychological tests within age-, gender-, and education-adjusted norms (>1.5 SD) and failure to meet the criteria for MCI; and (3) HIS score lower than 4.

### Fecal Sample Collection and DNA Extraction

Fresh fecal samples (approximately 400 mg) were collected 1 day before surgery, suspended in Longseegen stool storage solution according to the manufacturer’s protocol (Longsee Biomedical Corporation, Guangzhou, China) and stored at −80°C. Microbial DNA was extracted using the E.Z.N.A.^®^ soil DNA Kit (Omega Bio-tek, Norcross, GA, USA) according to the manufacturer’s protocols. The final DNA concentration and purity were determined by a Nanodrop 2000 UV-vis spectrophotometer (Thermo Scientific, Wilmington, USA), and DNA quality was checked by 1% agarose gel electrophoresis.

### 16S rRNA Gene Sequencing and Microbial Analysis

The V3-V4 hypervariable regions of the bacterial 16S ribosomal RNA (rRNA) gene were amplified with primers (338F and 806R) following the manufacturer’s protocol. Amplified PCR products were analyzed using 2% agarose gel electrophoresis, purified using an AxyPrep DNA Gel Extraction Kit (Axygen Biosciences, Union City, CA, USA), eluted by Tris-HCl, detected by 2% agarose electrophoresis, and quantified by QuantiFluor™ ST (Promega, USA). The purified amplified PCR fragments were constructed as PE 2300 libraries according to the Illumina MiSeq platform (Illumina, San Diego, USA) standard operating procedure. The Illumina MiSeq PE300 platform was used for sequencing.

The OTU raw data were filtered and processed, and the processed data were spliced and filtered to obtain effective data. Next, OTU clustering/denoising and species classification analysis were performed based on effective data to form species abundance spectra of OTUs and other species classification grades. The taxonomy of each 16S rRNA gene sequence was analyzed by the RDP Classifier algorithm (http://rdp.cme.msu.edu/) against the Silva (SSU128) 16S rRNA database using a confidence threshold of 70%.

### Enzyme-Linked Immunosorbent Assay

Blood samples were collected 1 day before surgery, and plasma was obtained by centrifugation at 2,000 × g for 15 min at 25°C and stored at –80°C. The plasma concentrations of CRP, interleukin-6 (IL-6), interleukin-10 (IL-10), interleukin-1β (IL-1β), and tumor necrosis factor alpha (TNF-α) were measured using an enzyme-linked immunosorbent assay (ELISA) kit (R&D SYSTEMS, USA). The concentrations of LPS, TJ protein claudin-1, and occludin were determined with an ELISA kit (HUAMEI, Wuhan, China).

### Statistical Analysis

Statistical analysis was performed using the SPSS 21.0 statistics program (SPSS, IBM, USA). Measurement data for three groups were compared with single factor analysis of variance (consistent with normal distribution and variance) or non-parametric statistics (not conforming to normal distribution or variance). The enumeration data were compared using the chi-square test.

All reads were deposited and grouped into operational taxonomic units (OTUs) at a sequence identity of 97%, and the taxonomic affiliation of the OTUs was determined with quantitative insights into microbial ecology (QIIME, version 2.0) against the Greengenes database version 135. The following downstream data analyses were conducted in R software. Pan and core OTUs were used to describe the changes in total species and core species as the sample size increased. Bacterial diversity was determined by α-diversity (Chao 1, Shannon’s index, InvSimpson index) and β-diversity (principal component analysis, PCA; principal coordinates analysis, PCoA). The Kruskal–Wallis test was performed to evaluate α-diversity among the different groups. ANOSIM was tested (analysis of similarities, a nonparametric test) for microbial community clustering (PCA, PCoA) using Bray-Curtis distance matrices. The linear discriminant analysis effect size (LEfSe) method was utilized to characterize the taxa with statistical significance and biological relevance. Taxa above 0.1% relative abundance were chosen for analysis and clarification. Paired comparisons were analyzed *via* the Wilcoxon rank-sum test. Correlation analysis was performed using Spearman’s rank tests. Based on Kyoto Encyclopedia of Genes and Genomes (KEGG) functional pathways, functional prediction of the intestinal microbiome was performed using the Phylogenetic Investigation of Communities by Reconstruction of Unobserved States test (PICRUSt), and functional differences among groups were assessed by Kruskal–Wallis test followed by Tukey-Kramer multiple comparisons. For all analyses, a value of P < 0.05 was considered to be significant.

## Results

### Participants’ Demographic and Clinical Characteristics

Participants’ demographic and clinical characteristics are summarized in **Table 1**. Neuropsychological assessment items were listed among the three groups (**Table 2**).

**Table 1:** Characteristics and perioperative data of the study population.

Characteristic	NC (n = 36)	SCD (n = 26)	aMCI (n = 18)	P value
Age (years)	70.78 ± 4.41	70.92 ± 3.55	71.22 ± 3.98	0.931
BMI (Kg/m^2^)	27.97 ± 3.10	26.94 ± 3.15	26.62 ± 3.94	0.288
Gender (male/female ratio)	10/26	7/19	2/16	0.893
Smoking (Yes/No ratio)	0/36	4/22	2/18	0.037^*^
Antibiotics	0	0	0	—
Food (Number)				0.291
HFHP	10	8	4	
HFLP	21	10	12	
LFHP	4	8	2	
LFLP	1	0	0	
ASA (Grade)				0.168
II	33	22	13	
III	3	4	5	
NYHA (Grade)				0.765
II	34	24	16	
III	2	2	2	
Hypertension (Number)	27	19	13	0.972
Diabetes (Number)	12	7	4	0.675
CHD (Number)	6	6	3	0.789
CVD (Number)	5	5	1	0.498
BP (mmHg)	104.2 ± 9.6	101.9 ± 10.8	102.3 ± 10.7	0.48
Rate (Times/min)	71.5 ± 8.9	69.4 ± 9.4	69.8 ± 12.6	0.54
SPO2 (%)	95.9 ± 1.9	92.3 ± 2.0	95.3 ± 1.9	0.252
WBC (10^9^/L)	5.82 ± 1.33	6.22 ± 1.36	6.49 ± 2.47	0.735
NEUT (10^9^/L)	3.39 ± 1.15	3.61 ± 0.80	3.55 ± 1.35	0.675
LYMPH (10^9^/L)	1.88 ± 0.63	1.96 ± 0.68	2.34 ± 1.19	0.634
LYMPH (%)	33.1 ± 10.4	31.3 ± 7.4	35.3 ± 9.4	0.38
Hb (g/dl)	13.2 ± 1.6	13.6 ± 2.0	12.2 ± 2.0	0.752
Glu (mmol/L)	6.6 ± 1.4	6.4 ± 0.9	7.9 ± 2.6	0.081
Lac (mmol/L)	1.1 ± 0.5	1.3 ± 0.5	1.0 ± 0.3	0.026^*^

BMI, Body mass index; Antibiotics value 0 meant that none of patients have used antibiotics within 3 months; HFHP, High fiber and high protein diet; HFLP, High fiber and low protein diet; LFHP, Low fiber and high protein diet; LFLP, Low fiber and low protein diet; ASA, American Standard Association; NYHA, New York Heart Association; CHD, Coronary heart disease; CVD, Cerebrovascular disease; BP, Blood pressure; SPO2, Saturation of peripheral oxygen; WBC, White blood cell; NEUT, Neutrophil; LYMPH, Lymphocyte; Hb, Hemoglobin; Glu, Blood glucose; Lac, Lactic. Measurement data of three groups were compared using single factor analysis of variance (consistent with normal distribution and variance) or non-parametric statistics (not conforming to normal distribution or variance). The enumeration data was compared using chi-square test. * shows P < 0.05.

**Table 2:** Differences in cognitive scale items between the three groups.

Item/Group	NC	SCD	aMCI	P Value
HAMD	0(0–10)	0(0–4)	0(0–20)	0.771
HAMA	0(0–10)	0(0–3)	0(0–34)	0.945
AVLT-H (S)	6.86 ± 1.588	5.27 ± 1.971	4.06 ± 1.798	<1e-04***
AVLT-H (L)	6.61 ± 1.500	4.81 ± 2.191	3.61 ± 1.720	<1e-04***
MoCA-B	25.25 ± 1.720	24.04 ± 2.490	18.50 ± 3.015	<1e-04***
BNT (unit)	3.97 ± 0.167	3.92 ± 0.272	3.39 ± 0.778	<1e-04***
VFT (unit)	17.00 ± 3.171	15.31 ± 3.696	14.00 ± 3.726	0.009**
STT-B (S)	171.97 ± 39.823	173.23 ± 45.921	220.06 ± 58.516	0.004***
CDT30	25.90 ± 2.792	25.65 ± 2.314	21.50 ± 3.974	<1e-04***
ADL	24.17 ± 7.225	24.62 ± 6.735	26.17 ± 9.218	0.374

HAMD, Hamilton's Depression Scale; HAMA, Hamilton's Anxiety Scale; AVLT-H (S), Auditory verbal learning test-Huashan version for short term; AVLT-H (L), Auditory verbal learning test-Huashan version for long term; MoCA-B, Montreal Cognitive Assessment-Basic; BNT, Bonston naming test; VFT, Verbal fluency test; STT-B, Shape trail test B; CDT30, Draw the clock test; ADL, Activity of Daily Living. BNT and VFT’s units were single, STT-B’s unit was second, and the other items’ units were scores. Skewed distribution data, such as depression and anxiety score data, were expressed by median, maximum, and minimum values, and the rest of normal distribution data were expressed by mean plus or minus standard deviation. And we applied non-parametric rank sum test in depression and anxiety score data analysis, and one way ANOVA analysis in the other neuropsychological assessment items. ** shows P < 0.01, *** shows P < 0.005.

### Alterations of Microbiomes in pAD Patients

In total, 4,496,945 sequencing reads were obtained in our sample, and the sequencing reads of each sample are shown in **Supplementary Table 1**. In addition, we described the sample base reads and the length of the sample sequence. The total number of base reads was 1,960,823,987, and the average length of the sample sequence was 436.2227439. Prior to the analysis, we annotated the valid OTU data by species taxonomy and counted the abundance information regarding annotated OTU results in each sample. We observed 1 domain, 1 kingdom, 33 phyla, 68 classes, 134 orders, 250 families, 588 genera, 1,092 species, and 1,932 OTUs in all samples. Pan analysis showed that with increasing sample number, the total OTU number of the NC group > SCD group > aMCI group (**Supplementary Figure 1A**). Core analysis indicated that with increasing sample number, the common OTU number of the NC group < SCD group < aMCI group (**Supplementary Figure 1B**).

**Figure 1:**
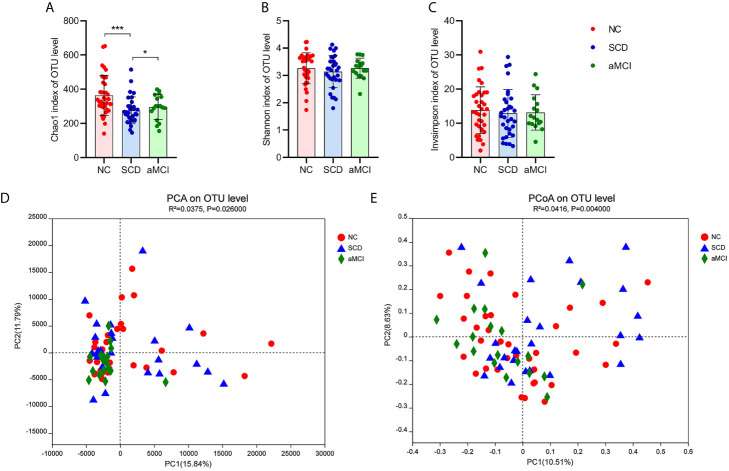
**(A–E)** Alpha and Beta diversity analysis in intestinal flora between different cognitive groups. **(A–C)** The X axis and the Y axis represent the groups and alpha diversity indices respectively. And the error bar means the standard deviation. Wilcoxon rank-sum test showed that the differences in chao1 index among the three groups were significant at the OTU level (*P-value < 0.05, ***P-value < 0.005, Wilcoxon rank sum test), which meant that the species richness of intestinal flora in the SCD group was lower than in the other two groups. Shannon and inverse Simpson indices were not different significantly among three groups, which indicated that the species evenness of intestinal flora was not obvious different between three groups (P-value > 0.05, Wilcoxon rank sum test). **(D, E)** The X axis and the Y axis represent two selected principal coordinate axes, and the percentage represent the interpretation value of the principal coordinate axis for the difference in sample composition. Each point represents a sample, whereas red circle, blue triangle, and green square represent the NC, SCD, and aMCI groups respectively. The closer the two sample points are, the more similar the species composition of the two samples is. Beta-diversity analysis results showed that the species composition in three groups was obvious different from each other. PCA and PCoA analysis both indicated that ANOSIM test results had a significant P value (PCA analysis: R^2^ = 0.0375, P = 0.026; PCoA analysis: R^2^ = 0.0416, P = 0.004).

There was a significantly lower degree of intraindividual diversity, as measured by the Chao1 diversity index (P = 0.004; **Figure 1A**), in SCD patients. However, the Shannon index and inverse Simpson index, indicating species diversity and evenness, did not differ significantly among the three groups (P = 0.627, 0.627; **Figures 1B, C**).

Principal component analysis (PCA) and principal coordinates analysis (PCoA) at the OTU level were applied in the species diversity analysis among the three groups. PCA and PCoA both exhibited beta diversity, as the species diversity was clearly different among the three groups (PCA and PCoA based on the Bray-Curtis distance algorithm, ANOSIM R^2^ = 0.038, P = 0.026 and R^2^ = 0.042, P = 0.004; **Figures 1D, E**).

### Identification of Key Microbiomes for Differentiating pAD From NC

**Figure 2:**
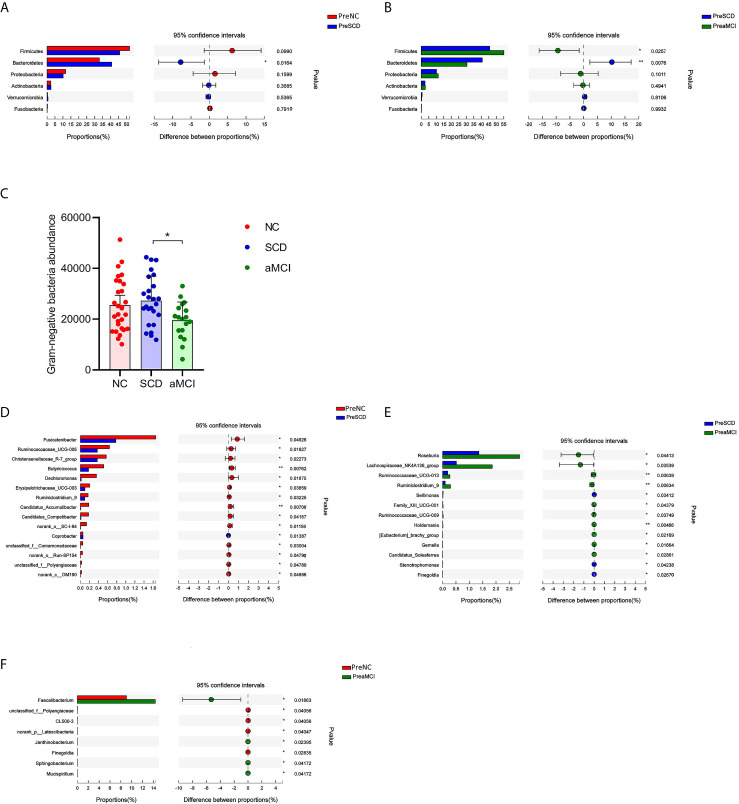
**(A–F)** Relative bacterial abundance and differences from phylum down to genus levels between different cognitive groups **(A–B)** The vertical axis is the different bacteria phyla and the horizontal axis is the proportion of phyla in the sample. The columns with red, blue, and green colors represent the NC, SCD, and aMCI groups, whereas the length of the columns represents the proportion of the phyla. And the interval between the left and the right bar represents the 95% confidence intervals. There were more *bacteroidetes* in the SCD group than in the other two groups, and less *Firmicutes* than in the aMCI group (*P-value < 0.05, **P-value < 0.01, Wilcoxon rank sum test). **(C)** The horizontal axis is the cognitive groups and the vertical axis is the abundance of Gram-negative bacteria at the family level. The columns with red, blue, and green colors represent the NC, SCD, and aMCI groups respectively. Wilcoxon rank sum test showed that the abundance of Gram-negative bacteria at the family level was higher in the SCD group than that in the aMCI group (*P-value = 0.047, Wilcoxon rank sum test). **(D–F)** The vertical axis is the different bacteria genera and the horizontal axis is the proportion of genera in the sample. The columns with red, blue, and green colors represent the NC, SCD, and aMCI groups, whereas the length of the columns represents the proportion of the genera. And the interval between the left and the right bar represents the 95% confidence intervals. Wilcoxon rank-sum test shows that the differences in relative abundance of gut bacteria among the three groups were significant at the genus level (*P-value < 0.05, **P-value < 0.01, ***P-value < 0.005, Wilcoxon rank sum test).

To determine the composition of the gut microbiota from the phylum to genus levels in the NC, SCD, and aMCI groups, the changes among these microbes were analyzed *via* LEfSe. *Bacteroidetes* and *Firmicutes* were the most dominant phyla among the three groups. The relative abundance of *Bacteroidetes* was higher in the SCD group than in the other two groups (40.7 *vs.* 30.5 *vs.* 32.9%, P = 0.016 and 0.008; **Figures 2A, B**). However, *Firmicutes* were less abundant in the SCD group than in the aMCI group (45.7 *vs.* 55.1%, P = 0.026; **Figure 2B**). Furthermore, the *Firmicutes/Bacteroidetes* (F/B) ratio (F/B = 1.12) was lower in the SCD group than in the NC (F/B = 1.58) and aMCI (F/B = 1.81) groups (P = 0.017).

The Gram-negative bacteria that differed significantly at the family level among the three groups are listed in **Table 3**. Among these bacteria, the *Christensenellaceae* family belonging to *Firmicutes* was detected at lower levels in the SCD and aMCI groups than in the NC group (P = 0.039; **Table 3**). At the family level, the total abundance of proinflammatory Gram-negative bacteria (54.00 ± 16.31%) was higher in the SCD group than in the aMCI group (43.41 ± 15.36%, P = 0.047; **Table 3** and **Figure 2C**) but did not differ from that observed in the NC group (47.63 ± 18.25%). At the genus level, the Gram-negative bacterium *Coprobacter* was more abundant in the aMCI group than in the other two groups (P = 0.035; **Table 4**).

**Table 3:** Abundance of Gram-negative bacteria families between different cognitive groups.

Gram-negative bacteria family/Group/Abundance	NC	SCD	aMCI	Corrected P value
	Mean±Sd	Mean±Sd	Mean±Sd	
f_Enterobacteriaceae	3906.828 ± 5162.281	4329.840 ± 6802.138	4331.167 ± 5463.394	0.608
f_Bacteroidaceae	11606.621 ± 7432.423	12667.880 ± 7404.790	10461.889 ± 7060.471	0.547
f_Rikenellaceae	681.828 ± 883.525	312.280 ± 393.213	413.444 ± 413.672	0.094
f_Acidaminococcaceae	361.690 ± 659.419	428.440 ± 423.626	351.778 ± 526.154	0.281
f_Christensenellaceae	412.931 ± 509.903	67.680 ± 161.526	84.278 ± 248.961	0.039*
f_Prevotellaceae	4106.000 ± 7954.082	4552.600 ± 8914.859	1172.500 ± 2563.799	0.588
f_Veillonellaceae	1163.828 ± 1335.407	673.880 ± 857.377	838.167 ± 1362.374	0.503
f_Desulfovibrionaceae	71.793 ± 79.112	71.640 ± 81.995	40.611 ± 30.805	0.688
f_Fusobacteriaceae	28.586 ± 128.120	82.800 ± 371.677	76.889 ± 316.546	0.818
f_Alcaligenaceae	0.759 ± 1.976	0.120 ± 0.332	0.111 ± 0.471	0.055
f_porphyromonadaceae	3121.724 ± 4593.482	4071.040 ± 5112.378	1751.722 ± 1894.365	0.212
Total Gram-negative bacteria families	25462.586 ± 10339.181	27258.200 ± 9642.427	19522.556 ± 7200.040	0.047*

The unit of the mean was abundance; Sd meant standard deviation. The Gram-negative bacteria families different significantly among three groups were listed in the above table. We applied non-parametric rank sum test in the analysis of abundance of Gram-negative bacteria families between three groups. * shows P < 0.05.

**Table 4:** Relative abundance of intestinal microbiota genera between different cognitive groups.

Microbial genera/Group/Relative abundance	NC	SCD	aMCI	Corrected P Value
	Mean±Sd	Mean±Sd	Mean±Sd	
*g__Ruminococcaceae_UCG-013*	0.2378 ± 0.3955	0.193 ± 0.6476	0.2732 ± 0.2754	0.028^*^
*g__Fusicatenibacter*	1.657 ± 2.064	0.7793 ± 0.913	2.733 ± 7.116	0.049^*^
*g__Ruminococcaceae_UCG-005*	0.6401 ± 1.181	0.3737 ± 0.8784	0.2902 ± 0.5211	0.049^*^
*g__Ruminiclostridium_9*	0.1691 ± 0.2093	0.0975 ± 0.127	0.296 ± 0.5229	0.013^*^
*g__Butyricicoccus*	0.5126 ± 1.14	0.1799 ± 0.2334	0.2513 ± 0.3014	0.028^*^
*g__Coprobacter*	0.05631 ± 0.1127	0.05759 ± 0.2399	0.2138 ± 0.6669	0.035^*^
*g__Christensenellaceae_R-7_group*	0.5694 ± 1.106	0.3725 ± 1.037	0.2522 ± 0.5949	0.023^*^
*g__Erysipelotrichaceae_UCG-003*	0.2056 ± 0.2826	0.09964 ± 0.1271	0.2397 ± 0.3314	0.039^*^
*g__Faecalibacterium*	9.043 ± 7.491	10.75 ± 9.598	14.34 ± 9.736	0.019^*^
*g__Lachnospiraceae_NK4A136_group*	0.6759 ± 1.025	0.5161 ± 0.8467	1.857 ± 3.991	0.035^*^
*g__Roseburia*	2 ± 3.403	1.357 ± 2.279	2.886 ± 3.561	0.044^*^

g_indicated genus. The unit of the mean was relative abundance (%); Sd meant standard deviation. The intestinal microbiota genera different significantly among three groups were listed in the above table. We applied non-parametric rank sum test in the analysis of relative abundance of intestinal microbiota genera between three groups. * shows P < 0.05.

LEfSe analysis showed that anti-inflammatory SCFA-producing bacteria at the genus level could differentiate the SCD group from either the NC or aMCI group. The SCFA-producing bacteria that differed significantly among the three groups are listed in **Table 4**. The relative abundance levels of six genera of SCFA-producing bacteria (*Fusicatenibacter*, *Ruminococcaceae_UCG-005*, *Christensenellaceae_R-7_group*, *Butyricicoccus*, *Erysipelotrichaceae_UCG-003*, and *Ruminiclostridium_9*) were lower in the SCD group than in the NC group (P < 0.05, **Table 4** and **Figure 2D**). The relative abundance levels of four genera of SCFA-producing bacteria (*Roseburia*, *Lachnospiraceae_NK4A136_group*, *Ruminococcaceae_UCG-013*, and *Ruminiclostridium_9*) were lower in the SCD group than in the aMCI group (P < 0.05, **Table 4** and **Figure 2E**). However, the levels of *Faecalibacterium* of SCFA-producing bacteria were lower in the NC group than in the aMCI group (P = 0.019, **Table 4** and **Figure 2F**).

### Predictive Function Analysis of the Microbiome in the pAD Group and NC Group

KEGG functional orthologs were predicted by PICRUSt. As shown in **Supplementary Table 2**, there was extensive communication between the gut microbiota and the patients in this study, including cellular processes, environmental information processing, genetic information processing, metabolism, and organismal systems. Specifically, seven functional orthologs were observed to be lower in the SCD group, and nine were observed to be lower in the aMCI group, as determined using the Level 2 KEGG pathways, compared with the NC group. The lower functional orthologs were replication and repair, amino acid metabolism, energy metabolism, lipid metabolism, metabolism of cofactors and vitamins, xenobiotic biodegradation and metabolism, and the immune system in the SCD group. Additionally, the aMCI group’s lower orthologs were replication and repair, energy metabolism, glycan biosynthesis and metabolism, lipid metabolism, metabolism of cofactors and vitamins, xenobiotic biodegradation and metabolism, the immune system, and the nervous system. Compared to the SCD group, the aMCI group exhibited a reduction in glycan biosynthesis and metabolism (P = 0.045).

**Figure 3:**
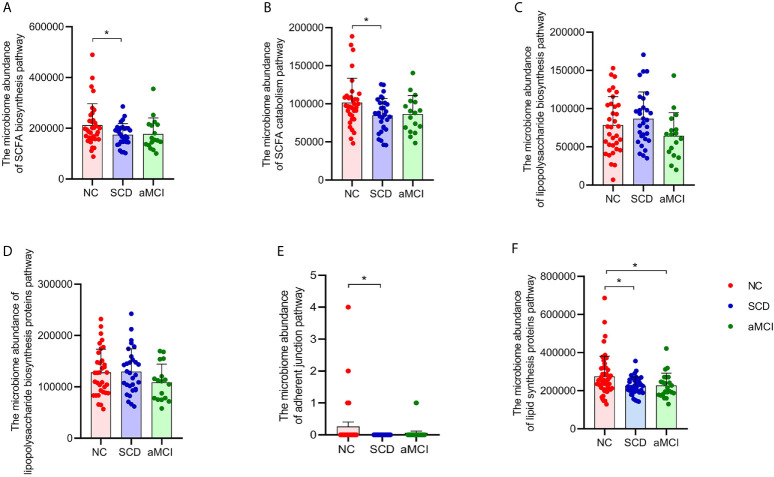
**(A–F)** Prediction analysis in intestinal flora between different cognitive groups. PICRUST compared the KEGG database to get the microbiome abundance of each metabolic pathway at KEGG pathway level 3. The Y axis and the X axis represent the intestinal abundance of each metabolic pathway at KEGG pathway level 3 and groups respectively, and the error bar means the standard deviation. The columns with red, blue, and green colors represent the NC, SCD, and aMCI groups respectively. Wilcoxon rank sum test showed that there were higher abundance of three metabolic pathways in NC group than those in SCD group (*P-value < 0.05, Wilcoxon rank sum test), including short-chain fatty acid biosynthesis (KEGG pathway id: ko00061; **(A)** and catabolism (KEGG pathway id: ko00071; **(B)** and adherent junction (KEGG pathway id: ko04520; **(E)**. There was insignificant difference between three cognitive groups in lipopolysaccharide biosynthesis (KEGG pathway id: ko00540; **(C)** and lipopolysaccharide biosynthesis proteins (KEGG pathway id: ko00541; **(D)** (*P-value < 0.05, Wilcoxon rank sum test). And Lipid biosynthesis proteins (KEGG pathway id: ko00537; **(F)** were lower in SCD and aMCI group compared with NC group (P = 0.018 and 0.027; Wilcoxon rank sum test).

More progressive and specific functional orthologs were predicted using KEGG pathway level 3. For example, fatty acid catabolism and biosynthesis were less frequently seen in the SCD group (P = 0.017 and 0.022; **Figures 3A, B**). However, lipopolysaccharide biosynthesis and lipopolysaccharide biosynthesis proteins did not differ significantly among the three groups (P > 0.05, **Figures 3C, D**). There were also fewer pathways involved in adherent junctions in the SCD group than in the NC group (P = 0.035; **Figure 3E**). Lipid biosynthesis proteins were lower in the SCD and aMCI groups than in the NC group (P = 0.018 and 0.027; **Figure 3F**).

### Correlation Analysis of Environmental Factors

**Figure 4:**
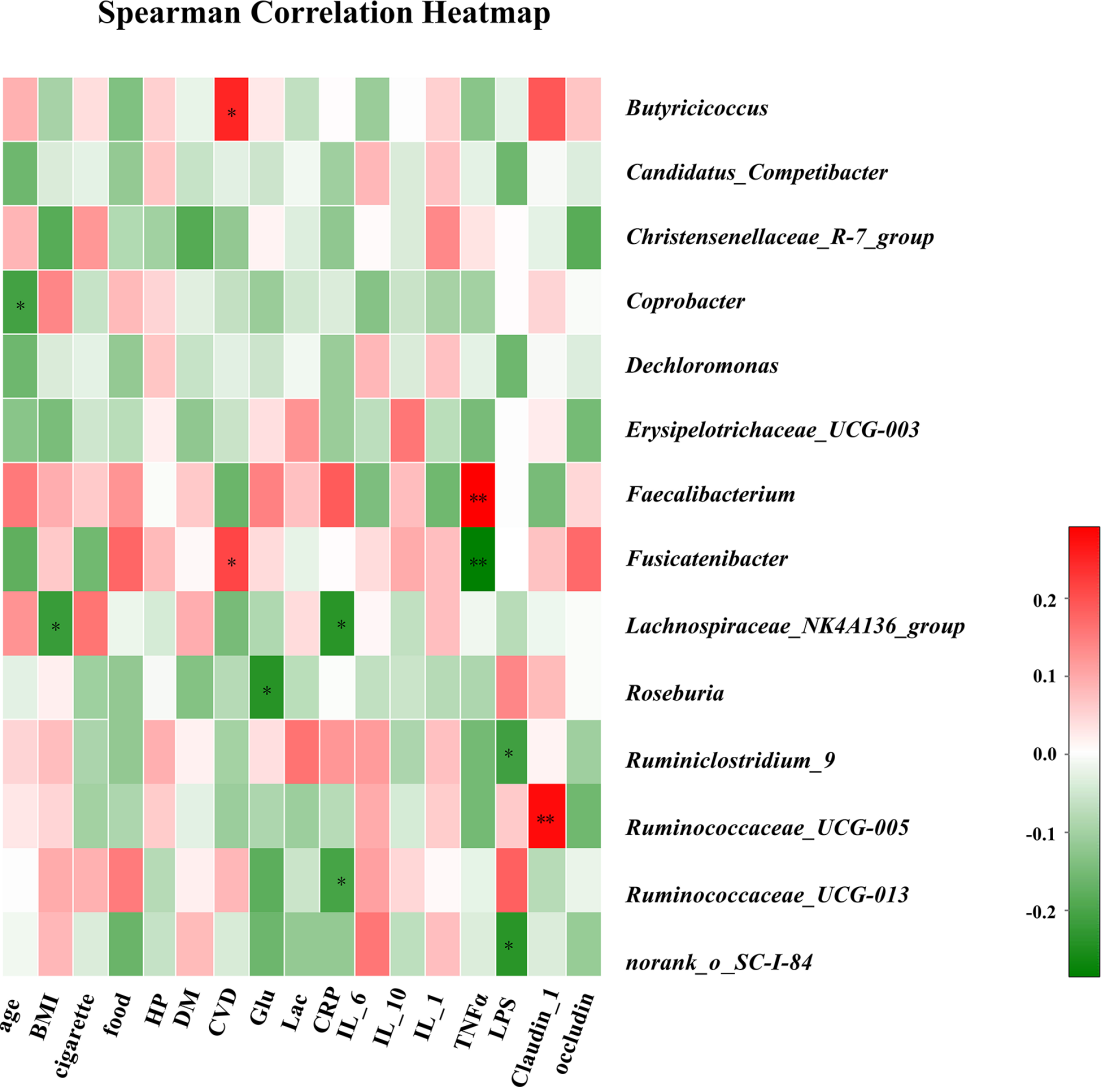
Correlation analysis of clinical parameters and gut microbiota. The association between 17 clinical parameters and significant bacterial genera with altered abundances among the three groups is estimated using the heat map of Spearman’s correlation analysis. Color intensity represents the magnitude of correlation. Red, positive correlations; Green, negative correlations. P-value < 0.05, **P-value < 0.01.

The associations between clinical parameters and different genera of gut bacteria among the three groups are shown in the heat-map in **Figure 4**. The levels of *Lachnospiraceae_NK4A136_group* were negatively correlated with BMI (r = −0.22), while CVD was positively correlated with *Butyricicoccus* and *Fusicatenibacter* (r = 0.25 and 0.21). Plasma glucose concentration was negatively correlated with *Roseburia* (r = −0.24). In addition, CRP levels were negatively correlated with *Lachnospiraceae_NK4A136_group* and *Ruminococcaceae_UCG-013* (r = −0.24 and −0.20). The TNF-α levels were positively correlated with *Faecalibacterium* (r = 0.29) and negatively correlated with *Fusicatenibacter* (r = −0.28). The LPS concentration in blood was negatively correlated with *Ruminiclostridium_9* (r = −0.21), and the level of claudin-1 was positively correlated with *Ruminococcaceae_UCG-005* (r = 0.28).

### Plasma Intestinal Linker Protein Levels and LPS and CRP Levels Were Elevated in pAD Patients

**Figure 5:**
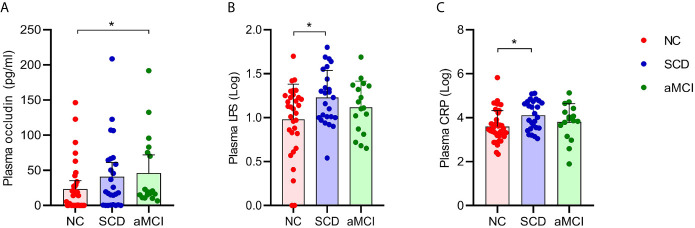
**(A–C)** Analysis of plasma factors levels between NC and pAD patients. The Y axis represents the concentration of plasma occludin, Log_10_LPS, and Log_10_CRP. The X axis represents the groups and the error bar means standard deviation. Wilcoxon rank sum test shows a significant difference in the plasma occludin levels between the aMCI group and the NC group **(A)** (*P-value < 0.05, Wilcoxon rank sum test). Wilcoxon rank sum test shows that the plasma Log_10_LPS and Log_10_CRP levels in the SCD group are significantly higher than those in the NC groups **(B, C)**.

The average plasma occludin levels in the aMCI group were almost twofold higher than those in the NC group (P = 0.039; **Table 5** and **Figure 5A**). However, plasma claudin-1 levels did not differ significantly (P = 0.681; **Table 5**) among the three groups.

**Table 5:** Levels of serum cytokines among three groups.

Cytokines/Group	NC	SCD	aMCI	P Value
	Mean ± Sd	Mean ± Sd	Mean ± Sd	
**CRP (ng/ml)**	27176.180 ± 109205.142	31194.959 ± 36097.585	36097.585 ± 32497.086	0.012^*^
IL-6 (pg/ml)	35.922 ± 129.500	39.523 ± 122.614	15.106 ± 27.0734	0.667
IL-10 (pg/ml)	16.831 ± 23.831	18.105 ± 33.906	24.661 ± 36.159	0.425
IL-1β (pg/ml)	11.375 ± 76.437	13.406 ± 75.059	0.009 ± 5.256	0.693
TNF-α (pg/ml)	18.171 ± 68.764	59.243 ± 144.528	17.596 ± 54.863	0.379
**Occludin (pg/ml)**	23.130 ± 35.685	40.968 ± 50.108	46.233 ± 51.765	0.039^*^
Claudin-1 (pg/ml)	13.452 ± 24.955	35.691 ± 69.372	13.705 ± 30.912	0.681
**LPS (pg/ml)**	31.255 ± 61.445	32.075 ± 33.256	40.655 ± 67.164	0.034^*^

CRP, C-reactive protein; IL, interleukin; TNF-α, tumor necrosis factor α; LPS, lipopolysaccharide. All cytokines data were skewed distribution data and displayed as mean plus or minus standard deviation. We applied non-parametric rank sum test in cytokines data analysis between different cognitive groups. * shows P < 0.05.

The plasma LPS levels in the SCD group were higher than those in the NC group (P = 0.034; **Table 5** and **Figure 5B**). The plasma LPS level in the aMCI group was higher than that in the NC group, but there was no significant difference between them (P = 0.200).

Plasma CRP levels were higher in the SCD group than in the NC group (P = 0.012; **Table 5** and **Figure 5C**). There was higher plasma CRP in the aMCI group than in the NC group, but the difference was not significant (P = 1.000). The serum IL-6, IL-10, IL-1β, and TNF-α levels did not differ significantly among the three groups (P > 0.05; **Table 5**).

### Correlations Between Gram-Negative Bacteria, SCFA-Producing Bacteria, and Tight Junction Proteins, Inflammation-Related Factors, and Neuropsychological Assessment of pAD Groups

**Figure 6:**
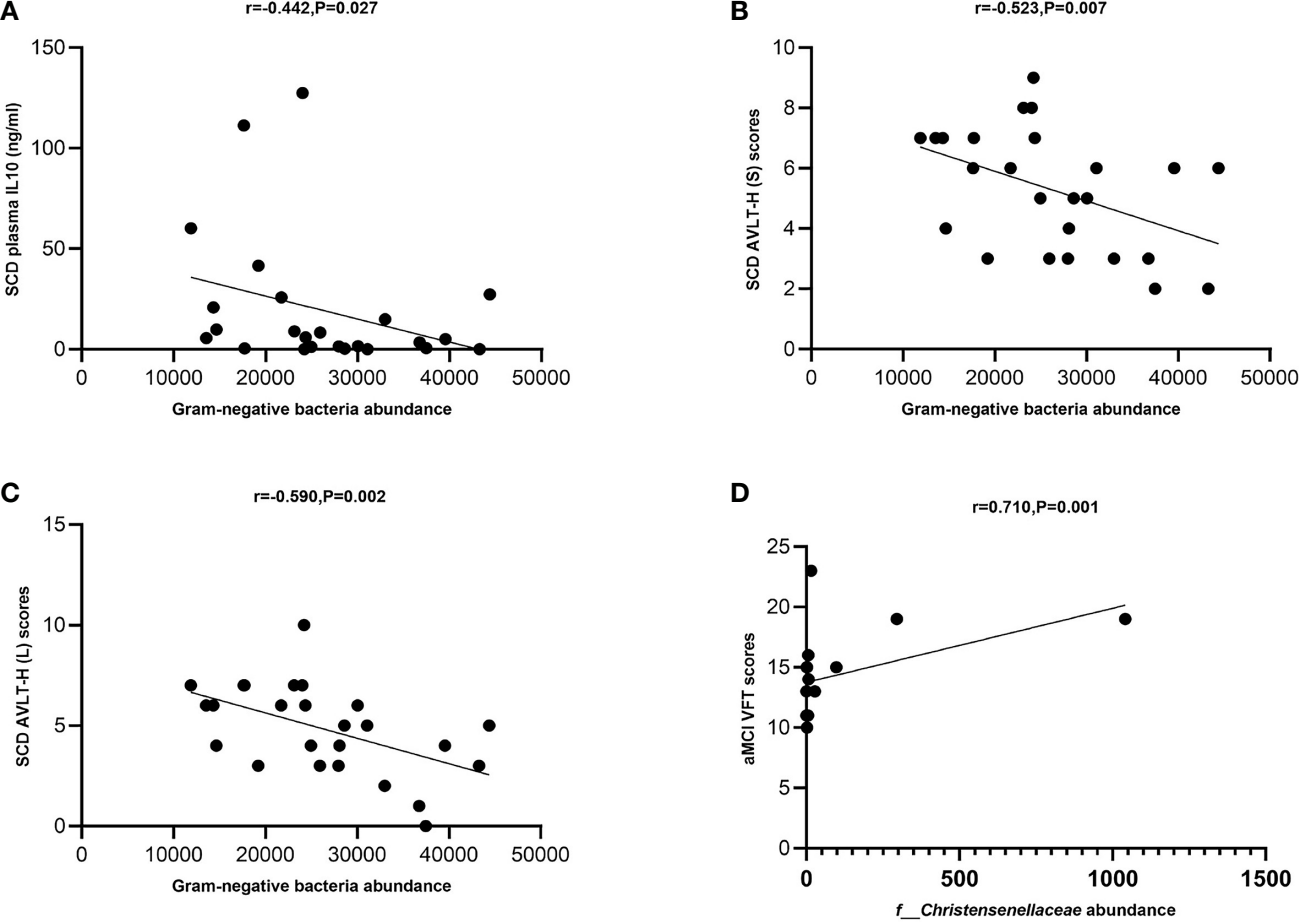
**(A–D)** Spearman’s correlation analysis between Gram-negative bacteria and inflammatory factors and neuropsychological assessment of pAD groups. **(A)** Spearman’s correlation analysis outcome shows that the levels of total Gram-negative bacteria were negatively correlated with plasma IL-10 level in the SCD group (Spearman correlation r = −0.442, P = 0.027; Linear regression equation: Y = −0.001195*X + 49.42, P = 0.1235). **(B, C)** The levels of total Gram-negative bacteria were negatively correlated with AVLT-H (S) and AVLT-H (L) respectively in the SCD group (Spearman correlation r = −0.523, P = 0.007 and r = −0.590, P = 0.002; Linear regression equation: Y = −9.806e-005*X + 7.872 and Y = −0.0001264*X + 8.162, P = 0.0244 and 0.0079). **(D)** The abundance of the *Christensenellaceae* family was positively with VFT in the aMCI group (Spearman correlation r = 0.710, P = 0.001; Linear regression equation: Y = 0.006150*X + 13.75, P = 0.0811).

The levels of total Gram-negative bacteria were negatively correlated with plasma IL-10 levels in the SCD group (Spearman correlation r = −0.442, P = 0.027; **Figure 6A**). The levels of total Gram-negative bacteria were negatively correlated with AVLT-H (S) and AVLT-H (L) in the SCD group (Spearman correlation r = −0.523, P = 0.007 and r = −0.590, P = 0.002; **Figures 6B, C**). In the aMCI group, the abundance of the *Christensenellaceae* family was positively correlated with VFT (Spearman correlation r = 0.710, P = 0.001; **Figure 6D**).

**Figure 7:**
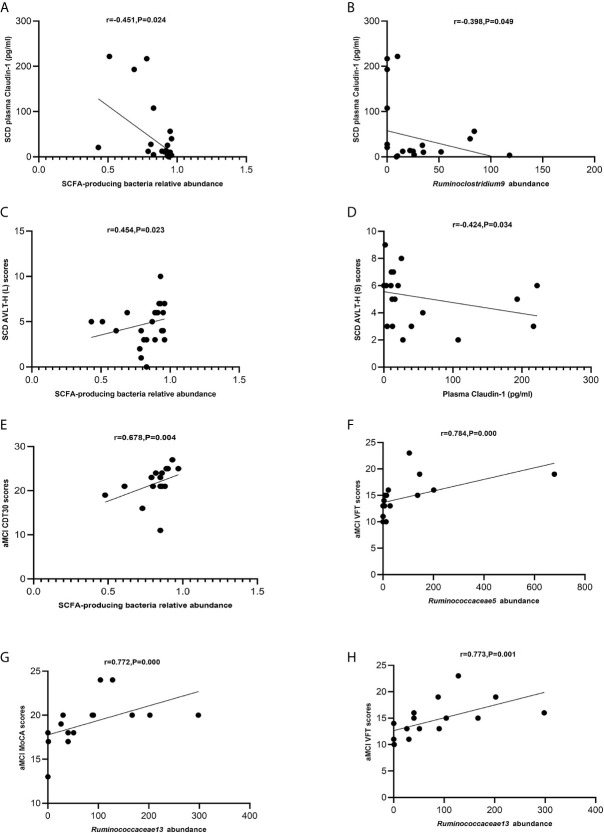
**(A–H)** Spearman’s correlation analysis between SCFA-producing bacteria and tight junction proteins and neuropsychological assessment of pAD groups. **(A)** Spearman’s correlation analysis outcome shows that the concentration of plasma claudin-1 was negatively correlated with the relative abundance of the SCFA-producing bacteria in the SCD group (r = −0.451, P = 0.024; Linear regression equation: Y = −224.0*X + 224.5, P = 0.0229). **(B)** Spearman’s correlation analysis outcome shows that the concentration of plasma claudin-1 was negatively correlated with the relative abundance of the SCFA-producing bacteria *Ruminoclostridium9* in the SCD group (r = −0.398, P = 0.049; Linear regression equation: Y = −0.5602*X + 57.64, P = 0.0962). **(C)** Spearman’s correlation analysis between the relative abundance of SCFA-producing bacteria and AVLT-H (L) scores in the SCD group (r = 0.454, P = 0.023; Linear regression equation: Y = 3.847*X + 1.610, P = 0.2345). **(D)** Spearman’s correlation analysis between the concentration of plasma caludin-1 and AVLT-H (S) scores in the SCD group (r = 0.424, P = 0.034; Linear regression equation: Y = −0.008021*X + 5.555, P = 0.1623). **(E)** Spearman’s correlation analysis between the relative abundance of SCFA-producing bacteria and CDT30 in the aMCI group (r = 0.678, P = 0.004; Linear regression equation: Y = 12.68*X + 11.33, P = 0.1302). **(F)** Spearman’s correlation analysis between the relative abundance of SCFA-producing bacteria *Ruminococcaceae5* and VFT scores in the aMCI group (r = 0.784, P= 0.000; Linear regression equation: Y = 0.01098*X + 13.63, P = 0.0359). **(G)** Spearman’s correlation analysis between the relative abundance of the SCFA-producing bacteria *Ruminococcaceae13* and MoCA-B scores in the aMCI group (r = 0.772, P = 0.000; Linear regression equation: Y = 0.01652*X + 17.76, P = 0.0368). **(H)** Spearman’s correlation analysis between the relative abundance of the SCFA-producing bacteria *Ruminococcaceae13* and VFT scores in the aMCI group (r = 0.773, P = 0.001; Linear regression equation: Y = 0.02425*X + 12.65, P = 0.019).

The levels of SCFA-producing bacteria were negatively correlated with those of claudin-1 in the SCD group (Spearman correlation r = −0.451, P = 0.024; **Figure 7A**). *Ruminoclostridium 9* was negatively correlated with claudin-1 in the SCD group (Spearman correlation r = −0.398, p = 0.049; **Figure 7B**). Moreover, the levels of SCFA-producing bacteria were positively correlated with those of AVLT-H(L) in the SCD group (Spearman correlation r = 0.454, P = 0.023; **Figure 7C**). In addition, claudin-1 was negatively correlated with AVLT-H(S) in the SCD group (Spearman correlation r = −0.424, P = 0.034; **Figure 7D**). In the aMCI group, the levels of SCFA-producing bacteria were positively correlated with those of CDT30 (Spearman correlation r = 0.678, P = 0.004; **Figure 7E**). The levels of *Ruminococcaceae 5* were positively correlated with VFT, and *Ruminococcaceae 13* was positively correlated with MoCA and VFT in the aMCI group (Spearman correlation r = 0.784, P = 0.000 and r = 0.772/0.773, P = 0.000/0.001; **Figures 7F–H**).

## Discussion

The pAD patients, who were scheduled for elective orthopedic surgery, exhibited gut microbiota dysbiosis and lower predicted functionality and intestinal barrier dysfunction compared with the matched NC patients. In pAD patients, especially SCD patients, gut microbiota dysbiosis was observed, including a reduction in gut microbiota α-diversity, an increased level of Gram-negative bacteria, and a decreased level of SCFA-producing bacteria in the intestine. Moreover, intestinal barrier dysfunction, low-grade systemic inflammation, and clinical parameters were associated with gut microbiota dysbiosis in pAD patients.

The fecal microbial diversity, as estimated by the Chao1 index, was lower in the SCD patients than in the NC patients in this study, which was in keeping with the findings of previous studies in both aMCI and AD patients (Vogt et al., 2017). The Chao1 index indicated gut microbiota species richness (Hughes et al., 2001), which reflected the stability and resilience of the gut microbiome (Fassarella et al., 2020). A lower Chao1 index results in a decreased level of functional redundancy and less effectiveness in using limiting resources, further resulting in unstable microbiota functions during perturbations and poor resilience (Fassarella et al., 2020). Although the exact effect of the reduced bacterial diversity in pAD has not been fully elucidated, it may predict these patients’ susceptibility to intestinal microbial dysbiosis (Li B. et al., 2019; Liu P. et al., 2019) and might play important roles in AD progression (Vogt et al., 2017).

In this study, the F/B ratio of gut microbiota was higher among aMCI patients than among NC and SCD patients, which was in keeping with the high F/B ratio of AD patients observed in a previous study (Zhuang et al., 2018) and might predict the onset and worsening of cognitive decline symptoms in humans (Ticinesi et al., 2019). However, NC patients had a higher F/B ratio than SCD patients, which was in keeping with the findings obtained in a previous study, in which a cognitively normal elderly population exhibited a high F/B ratio (Manderino et al., 2017). Thus, the relationship between the F/B ratio and cognitive function should be further assessed.

Gram-negative bacteria of the gut microbiota are enriched in aMCI and AD patients compared with NC patients in the Chinese cohort (Li B. et al., 2019; Liu P. et al., 2019). In our study, the total abundance of Gram-negative bacteria at the family level was highest in SCD patients. Specifically, the family *Christensenellaceae*, emerging as an important player in human health (Waters and Ley, 2019), was enriched in NC patients. To compare the capability of Gram-negative bacteria to biosynthesize LPS among the three groups of patients, PICRUST analysis was performed. The analysis found no discrepancy in functional microbiota profiles of the LPS biosynthesis pathway among the three groups. These results inferred that the elevation of plasma LPS in the SCD group may be primarily attributable to intestinal barrier dysfunction, which has been associated with the reduction in the levels of SCFA-producing microbiota (Perez-Pardo et al., 2019).

Compared to NC patients, the levels of six butyrate-producing microbiota genera (Louis and Flint, 2017) were decreased in SCD patients, which was in keeping with the findings of a previous study (Zhuang et al., 2018). Microbiota-derived butyrate plays an essential role in maintaining the integrity of the gut barrier by providing energy for the colonocytes, expressing tight junction proteins in colon epithelia and exhibiting anti‐inflammatory effects (Koh et al., 2016; Zheng et al., 2017). The decrease in SCFA-producing bacteria might further contribute to the progression of AD (Ising et al., 2019) with the reduction of butyrate, intestinal barrier disruption, and the translocation of endotoxin into the circulatory system (Perez-Pardo et al., 2019). The above results were in line with a gradual decrease in the levels of SCFA-producing microbiota in the progression of AD (Zhuang et al., 2018). Among these decreased butyrate-producing microbiota genera in the SCD group compared to the NC group, *Fusicatenibacter* in family *Lachnospiraceae*, which increases butyrate production in stool samples (Weis et al., 2019), maintains the integrity of the gut barrier and downgrades inflammation (Koh et al., 2016; Zheng et al., 2017), was also decreased in patients with cirrhosis, PD, ulcerative colitis, and Crohn’s disease (Takeshita et al., 2016; Jin et al., 2019; Weis et al., 2019; Qiu et al., 2020). The low abundance of *Ruminococcaceae_UCG-005* within the family *Ruminococcaceae* in SCD patients was in keeping with the findings of a report on patients with depression (Jiang et al., 2015). However, *Ruminococcaceae_UCG-005* was enriched in cerebral ischemic stroke patients (Li N. et al., 2019). The genus *Christensenellaceae_R-7_group* within family *Christensenellaceae*, was also observed less in the SCD patients, similar to the trend observed in the patients with hypertension compared to the control group (Calderón-Pérez et al., 2020). As a Gram-positive anaerobic *Clostridium* cluster IV bacterium, *Butyricicoccus* decreased in pAD patients compared to NC patients, which was in keeping with the change in gut microbiota observed in patients with Ulcerative Colitis and Crohn’s disease (Eeckhaut et al., 2013). However, the increase in the genus *Butyricicoccus* was associated with cognitive impairment in Chinese patients with Parkinson’s disease (Qian et al., 2018). Our study also showed that *Butyricicoccus* was positively associated with cerebrovascular disease, which was in keeping with the findings of another previous study (Li N. et al., 2019). A low abundance of *Erysipelotrichaceae_UCG-003* was observed in SCD patients compared to NC patients in our study, which was correlated with a high-fat diet (Parmentier-Decrucq et al., 2009), colorectal cancer (Chen et al., 2012), irritable bowel disease (IBD), and Crohn’s disease (Schaubeck et al., 2016), while a high abundance of *Erysipelotrichaceae_UCG-003* was observed in healthy aging cohorts (Singh et al., 2019). In addition, we found that the abundance of *Ruminiclostridium_9* within the family *Hungateiclostridiaceae* was lower in SCD patients than in aMCI and NC patients regardless of smoking, which was observed in healthy men who smoke and drink alcohol or in overweight and obese adults (Renbin et al., 2020; van Trijp et al., 2020). Recently, an animal study showed that *Ruminiclostridium_9* and seven other genera of butyrate-producing bacteria can improve systemic inflammation and intestinal permeability in aged mice fed a high-fat diet (Zhang et al., 2020). In our study, *Ruminiclostridium_9* was decreased and negatively correlated with blood LPS and claudin-1 in the SCD group. These results suggest that *Ruminiclostridium_9 is* possibly involved in maintaining intestinal barrier function in the elderly.

Except for the genus *Ruminiclostridium_9*, there were three high-level genera of butyrate-producing bacteria in the aMCI group compared with the SCD group. With the production of considerable amounts of butyrate, *Roseburia* within the family *Lachnospiraceae* may be important for the control of inflammatory processes, especially in the gut, which decrease PD, irritable bowel syndrome, obesity, and type 2 diabetes (Tamanai-Shacoori et al., 2017). However, in our study, a high abundance of *Roseburia* was detected in aMCI patients, suggesting that the high abundance of *Roseburia* was related to severe cognitive impairment. The abundance of *Lachnospiraceae_NK4A136_group* within family *Lachnospiraceae* was observed to improve gut barrier function in aging rats (Li J. et al., 2019). *Lachnospiraceae_NK4A136_group* was enriched in aMCI patients in our study, which was similar to the animal study (Gao et al., 2019) and opposite to findings obtained in dementia patients (Stadlbauer et al., 2020). *Ruminococcaceae_UCG-013*, belonging to the family *Ruminococcaceae*, was enriched in the aMCI group compared with the SCD group in our study and was negatively correlated with inflammation and T2DM (Wu et al., 2017; Wei et al., 2018). A high abundance of *Faecalibacterium* was detected in aMCI patients compared to NC patients, which was also reported in patients with chronic insomnia (Jiang et al., 2020). However, decreased *Faecalibacterium* was observed in several diseases (Jiang et al., 2015; Evans et al., 2017; Ferreira-Halder et al., 2017; Tamanai-Shacoori et al., 2017; Jiang et al., 2018), such as IBD, colorectal cancer, and bipolar disorder. *Coprobacter*, as a SCFA-producing bacteria, was more abundant in the aMCI group than in the other two groups in our study, which was similar to the alteration of gut microbiota observed in children with autism spectrum disorder (Liu S. et al., 2019). The above results indicated that the effect of some SCFA-producing microbiota on early cognitive decline has not been elucidated and warrants further study.

According to the PICRUSt analysis, amino acid metabolism, energy metabolism (fatty acid catabolism and biosynthesis), lipid metabolism (lipid biosynthesis proteins), metabolism of cofactors and vitamins, xenobiotic biodegradation and metabolism, immune system, and nervous system were lower in the pAD group than in the NC group. Microbiota-mediated signals can reach the brain *via* neural, endocrine, and immune communication pathways (Holzer and Farzi, 2014). The products of this metabolism are considered to be precursors of bacterial synthesis of short-chain fatty acids (SCFAs) (Wei et al., 2013), which play important roles in regulating neurological function (Haast and Kiliaan, 2015). Moreover, the reduction in metabolism of cofactors and vitamins, xenobiotic biodegradation, and metabolism could be related to the fact that pAD patients are characterized by hypometabolism (Cornejo-Pareja et al., 2020). Meanwhile, the fewer modules for the immune system observed in the pAD group suggested depletion of immune system modulators, which indicated that IM might regulate the activation of immune and neuroinflammation, further correlated with neurodegenerative diseases (Fung et al., 2017). Furthermore, there were fewer modules for adherent junctions in the SCD group than in the NC group. The abovementioned results indicated a possible treatment for pAD by regulating mediators in the microbe-gut-brain axis, such as SCFAs, lipids, and vitamins. However, functional analysis did not provide specific microbial information on functional changes, and further research is warranted.

In our study, plasma LPS levels were higher in SCD patients than in the other two groups. High and low plasma LPS levels can drive amyloid beta production and aggregation in the AD brain (Vutukuri et al., 2018). Moreover, the upregulation of plasma CRP in SCD patients is consistent with the findings of a previous study (Beydoun et al., 2018). A strong association between the elevation of plasma CRP and longitudinal cognitive decline has been detected, largely among older individuals (Beydoun et al., 2018). In correlation analysis, inflammatory factors, including CRP and TNF-α, were negatively correlated with *Ruminococcaceae_UCG-013*, *Lachnospiraceae_NK4A136_group*, and *Fusicatenibacter*, which indicated that lower levels of butyrate-producing bacteria are associated with elevated levels of LPS and proinflammatory factors (Zhao et al., 2017). Meanwhile, plasma claudin-1 was negatively correlated with the relative abundance of SCFA-producing microbiota. Plasma IL-10 was negatively correlated with the total abundance of Gram-negative proinflammatory bacteria at the family level in the SCD group, which was in keeping with the findings of a previous study (Li F. et al., 2019); (Li et al., 2020). There was higher plasma occludin in aMCI patients than in NC patients, and memory function was negatively related to plasma claudin-1 in SCD patients, possibly suggesting that loss of intestinal barrier integrity may be correlated with cognition.

We also observed associations between Gram-negative bacteria, SCFA-producing microbiota, and neurocognitive function. Decreases in executive, memory, and language functions discriminate pAD from normal aging (Valech et al., 2018). In this study, the total abundance of Gram-negative bacterial families was negatively correlated with memory in the SCD group. The abundance of the *Christensenellaceae* family was positively related to language function in the aMCI group. In addition, we observed that the total SCFA-producing microbiota was positively associated with episodic memory in the SCD group. Furthermore, total SCFA-producing flora and *Ruminococcaceae_UCG-005* and *Ruminococcaceae_UCG-013* were positively correlated with executive functioning, language function, and global cognition in the aMCI group. Neurocognitive impairment in aMCI patients is manifested by decreases in episodic memory, executive functioning, processing speed, visuospatial function, and global neurocognition (Kim et al., 2017). Determining the relationships among Gram-negative bacteria, SCFA-producing microbiota, and neurocognitive function may help to elucidate the origin and evolution of AD.

The results indicate that subjects with different levels of cognitive impairment have significant differences in microbiota composition and predicted functionality compared with age-matched subjects without cognitive impairment. The decrease in short-chain fatty acid (SCFA)-producing bacteria in pAD is clear. Thus, depletion of SCFAs might be employed as a potential biomarker of the early stages of cognitive impairment, but this possibility requires focused investigation in future studies. This study should be viewed in light of the following limitations. First, we did not investigate fecal SCFAs. Therefore, we are uncertain whether the decreased levels of SCFA-producing microbiota result in reduced levels of fecal SCFAs, possibly causing injury to the intestinal barrier. Second, the small number of samples that we studied could have been affected by individual differences in gut microbiota. This limitation can be addressed by performing additional studies with more participants.

## Data Availability Statement

The original contributions presented in the study are publicly available. This data can be found in NCBI: PRJNA690972.

## Ethics Statement

The studies involving human participants were reviewed and approved by the ethics committee of Xuanwu Hospital of Capital Medical University, 2018-048. The patients/participants provided their written informed consent to participate in this study. Written informed consent was obtained from the individual(s) for the publication of any potentially identifiable images or data included in this article.

## Author Contributions

MD, FL, HF, and TW designed and supervised the project. TW and HF obtained funding. SL provided samples and performed clinical diagnosis and treatment. MD and FL collected samples, extracted data, and performed statistical analysis. MD and FL drafted the manuscript. TW and HF revised the manuscript for important content. All authors contributed to the article and approved the submitted version.

## Funding

This work was supported by the Beijing Municipal Administration of Hospitals Clinical Medicine Development of Special Funding Support under Grant (ZYLX201818).

## Conflict of Interest

The authors declare that the research was conducted in the absence of any commercial or financial relationships that could be construed as a potential conflict of interest.

## Supplementary Material

Supplementary Figure 1
**(A, B)** OTU analysis of three groups. The horizontal axis represents the number of samples observed, and the vertical axis represents the total/core species of all samples under a grouping category. Pan OTU is the sum of the OTU contained in all samples, which is used to observe the increase of the total number of OTU as the number of samples increases. Core OTU refers to the number of common OTU in all samples, which is used to observe the decrease of the number of common OTU as the number of samples increases. Red, blue, and green curves represent the NC, SCD, and aMCI groups’ OTU analysis.

## References

[B1] Baars L. M. A. E. van Boxtel M. P. J. Visser P. J. Verhey F. R. J. Jolles J. (2008). O2-01-03: Is mild cognitive impairment a stable diagnostic entity? Alzheimer’s Dementia 4 (4), T131. 10.1016/j.jalz.2008.05.306

[B2] Beydoun M. A. Dore G. A. Canas J. A. Liang H. Beydoun H. A. Evans M. K.. (2018). Systemic Inflammation Is Associated With Longitudinal Changes in Cognitive Performance Among Urban Adults. Front. Aging Neurosci. 10, 313. 10.3389/fnagi.2018.00313 30356710 PMC6189312

[B3] Bonfili L. Cecarini V. Berardi S. Scarpona S. Suchodolski J. S. Nasuti C.. (2017). Microbiota modulation counteracts Alzheimer’s disease progression influencing neuronal proteolysis and gut hormones plasma levels. Sci. Rep. 7 (1), 2421–2426. 10.1038/s41598-017-02587-2 28546539 PMC5445077

[B4] Brown G. C. (2019). The endotoxin hypothesis of neurodegeneration. J. Neuroinflamm. 16 (1), 180. 10.1186/s12974-019-1564-7 PMC6744684 31519175

[B5] Calderón-Pérez L. Gosalbes M. J. Yuste S. Valls R. M. Pedret A. Llauradó E.. (2020). Gut metagenomic and short chain fatty acids signature in hypertension: a cross-sectional study. Sci. Rep. 10 (1), 6436. 10.1038/s41598-020-63475-w 32296109 PMC7160119

[B6] Cattaneo A. P. Cattane N. P. Galluzzi S. M. Provasi S. M. Lopizzo N. M. Festari C. M.. (2016). Association of brain amyloidosis with pro-inflammatory gut bacterial taxa and peripheral inflammation markers in cognitively impaired elderly. Neurobiol. Aging 49, 60–68. 10.1016/j.neurobiolaging.2016.08.019 27776263

[B7] Chen W. Liu F. Ling Z. Tong X. Xiang C. Moschetta A. (2012). Human intestinal lumen and mucosa-associated microbiota in patients with colorectal cancer. PloS One 7 (6), e39743. 10.1371/journal.pone.0039743 22761885 PMC3386193

[B8] Chen K. L. Xu Y. Chu A. Q. Ding D. Liang X. N. Nasreddine Z. S.. (2016). Validation of the Chinese Version of Montreal Cognitive Assessment Basic for Screening Mild Cognitive Impairment. J. Am. Geriatrics Soc. (JAGS) 64 (12), e285–e290. 10.1111/jgs.14530 27996103

[B9] Clausen M. R. Mortensen P. B. (1995). Kinetic studies on colonocyte metabolism of short chain fatty acids and glucose in ulcerative colitis. Gut 37 (5), 684–689. 10.1136/gut.37.5.684 8549946 PMC1382875

[B10] Cornejo-Pareja I. Ruiz-Limón P. Gómez-Pérez A. M. Molina-Vega M. Moreno-Indias I. Tinahones F. J. (2020). Differential Microbial Pattern Description in Subjects with Autoimmune-Based Thyroid Diseases: A Pilot Study. J. Personalized Med. 10 (4), 192. 10.3390/jpm10040192 PMC7712884 33114469

[B11] Eeckhaut V. Machiels K. Perrier C. Romero C. Maes S. Flahou B.. (2013). Butyricicoccus pullicaecorum in inflammatory bowel disease. Gut 62 (12), 1745–1752. 10.1136/gutjnl-2012-303611 23263527

[B12] Evans S. J. Bassis C. M. Hein R. Assari S. Flowers S. A. Kelly M. B.. (2017). The gut microbiome composition associates with bipolar disorder and illness severity. J. Psychiatr. Res. 87, 23–29. 10.1016/j.jpsychires.2016.12.007 27988330 PMC5336480

[B13] Fassarella M. Blaak E. E. Penders J. Nauta A. Smidt H. Zoetendal E. G. (2020). Gut microbiome stability and resilience: elucidating the response to perturbations in order to modulate gut health. Gut 70, 595–605. 10.1136/gutjnl-2020-321747 33051190

[B14] Ferreira-Halder C. V. Faria A. V. D. S. Andrade S. S. (2017). Action and function of Faecalibacterium prausnitzii in hD ealth and disease. *Baillière’s best practice & research* . Clin. Gastroenterol 31 (6), 643–648. 10.1016/j.bpg.2017.09.011 29566907

[B15] Fung T. C. Olson C. A. Hsiao E. Y. (2017). Interactions between the microbiota, immune and nervous systems in health and disease. Nat. Neurosci. 20 (2), 145–155. 10.1038/nn.4476 28092661 PMC6960010

[B16] Gao H. Jiang Q. Ji H. Ning J. Li C. Zheng H. (2019). Type 1 diabetes induces cognitive dysfunction in rats associated with alterations of the gut microbiome and metabolomes in serum and hippocampus. *Biochimica et biophysica acta* . Mol. Basis Dis. 1865 (12), 165541. 10.1016/j.bbadis.2019.165541 31472216

[B17] Govindarajan N. Agis-Balboa R. C. Walter J. Sananbenesi F. Fischer A. (2011). Sodium butyrate improves memory function in an Alzheimer’s disease mouse model when administered at an advanced stage of disease progression. J. Alzheimer’s Dis. 26 (1), 187–197. 10.3233/JAD-2011-110080 21593570

[B18] Guo Q. Sun Y. Yu P. (2007). Norm of Auditory Verbal Learning Test in the Normal Aged in China Community. Chinese J. Clin. Psych. 15, 132–134, 141.

[B19] Guo Q. Fu J. H. Yuan J. Zhao Q. Cao X. Hong Z. (2008). A study of validity of a new scoring system of clock drawing test. Chin. J. Neurol. 41, 234–237. 10.16128/j.cnki.1005-3611.2007.02.009

[B20] Haast R. A. M. Kiliaan A. J. (2015). Impact of fatty acids on brain circulation, structure and function. Prostaglandins Leukotrienes Essential Fatty Acids 92, 3–14. 10.1016/j.plefa.2014.01.002 24485516

[B21] He Y. L. Zhai G. Y. Xiong X. Y. Chi Y. F. Zhang M. Y. Zhang M. Q. (1990). Assessment of activities of daily living in the elderly. Chi J. Gerontol. 10, 266–269.

[B22] Holota Y. Dovbynchuk T. Kaji I. Vareniuk I. Dzyubenko N. Chervinska T.. (2019). The long-term consequences of antibiotic therapy: Role of colonic short-chain fatty acids (SCFA) system and intestinal barrier integrity. PloS One 14 (8), e220642. 10.1371/journal.pone.0220642 PMC6705842 31437166

[B23] Holzer P. Farzi A. (2014). Neuropeptides and the microbiota-gut-brain axis. Adv. Exp. Med. Biol. 817, 195–219. 10.1007/978-1-4939-0897-4_9 24997035 PMC4359909

[B24] Hughes J. B. Hellmann J. J. Ricketts T. H. Bohannan B. J. (2001). Counting the uncountable: statistical approaches to estimating microbial diversity. Appl. Environ. Microbiol. 67 (10), 4399–4406. 10.1128/aem.67.10.4399-4406.2001 11571135 PMC93182

[B25] Iliff L. Zilhka E. Boulay G. McAllister V. Marshall J. Russell R.. (1975). Cerebral Blood Flow in Dementia. Arch. Neurol. 32, 632–637. 10.1001/archneur.1975.00490510088009 1164215

[B26] Ising C. Venegas C. Zhang S. Scheiblich H. Schmidt S. V. Vieira-Saecker A.. (2019). NLRP3 inflammasome activation drives tau pathology. Nature 575 (7784), 669–673. 10.1038/s41586-019-1769-z 31748742 PMC7324015

[B27] Jessen F. Amariglio R. E. van Boxtel M. Breteler M. Ceccaldi M. Chetelat G.. (2014). A conceptual framework for research on subjective cognitive decline in preclinical Alzheimer’s disease. Alzheimers Dement. 10 (6), 844–852. 10.1016/j.jalz.2014.01.001 24798886 PMC4317324

[B28] Jiang H. Ling Z. Zhang Y. Mao H. Ma Z. Yin Y.. (2015). Altered fecal microbiota composition in patients with major depressive disorder. Brain Behav. Immun. 48, 186–194. 10.1016/j.bbi.2015.03.016 25882912

[B29] Jiang H. Zhang X. Yu Z. Zhang Z. Deng M. Zhao J.. (2018). Altered gut microbiota profile in patients with generalized anxiety disorder. J. Psychiatr. Res. 104, 130–136. 10.1016/j.jpsychires.2018.07.007 30029052

[B30] Jiang M. Wang T. Zhang B. Wang D. Liu X. Tang H.. (2020). Gut Microbiota Changes and Their Relationship with Inflammation in Patients with Acute and Chronic Insomnia. Nat. Sci. Sleep 12, 895–905. 10.2147/NSS.S271927 33177907 PMC7652227

[B31] Jin M. Kalainy S. Baskota N. Chiang D. Deehan E. C. McDougall C.. (2019). Faecal microbiota from patients with cirrhosis has a low capacity to ferment non-digestible carbohydrates into short-chain fatty acids. Liver Int. 39 (8), 1437–1447. 10.1111/liv.14106 30919578

[B32] Kim J. Na H. K. Byun J. Shin J. Kim S. Lee B. H.. (2017). Tracking Cognitive Decline in Amnestic Mild Cognitive Impairment and Early-Stage Alzheimer Dementia: Mini-Mental State Examination versus Neuropsychological Battery. Dementia Geriatric Cogn. Disord. 44 (1-2), 105–117. 10.1159/000478520 28768247

[B33] Koh A. De Vadder F. Kovatcheva-Datchary P. Bäckhed F. (2016). From Dietary Fiber to Host Physiology: Short-Chain Fatty Acids as Key Bacterial Metabolites. Cell 165 (6), 1332–1345. 10.1016/j.cell.2016.05.041 27259147

[B34] Li B. He Y. Ma J. Huang P. Du J. Cao L.. (2019). Mild cognitive impairment has similar alterations as Alzheimer’s disease in gut microbiota. Alzheimers Dement. 15 (10), 1357–1366. 10.1016/j.jalz.2019.07.002 31434623

[B35] Li N. Pang B. Liu G. Zhao X. Xu X. Jiang C.. (2020). Lactobacillus rhamnosus from human breast milk shows therapeutic function against foodborne infection by multi-drug resistant Escherichia coli in mice. Food Funct. 11 (1), 435–447. 10.1039/c9fo01698h 31829373

[B36] Li F. Wang M. Wang J. Li R. Zhang Y. (2019). Alterations to the Gut Microbiota and Their Correlation With Inflammatory Factors in Chronic Kidney Disease. Front. Cell Infect. Microbiol. 9, 206. 10.3389/fcimb.2019.00206 31245306 PMC6581668

[B37] Li J. Wu T. Li N. Wang X. Chen G. Lyu X. (2019). Bilberry anthocyanin extract promotes intestinal barrier function and inhibits digestive enzyme activity by regulating the gut microbiota in aging rats. Food Funct. 1 (1), 333–343. 10.1039/c8fo01962b 30575836

[B38] Li N. Wang X. Sun C. Wu X. Lu M. Si Y.. (2019). Change of intestinal microbiota in cerebral ischemic stroke patients. BMC Microbiol. 19 (1), 191. 10.1186/s12866-019-1552-1 31426765 PMC6700817

[B39] Liu P. Wu L. Peng G. Han Y. Tang R. Ge J.. (2019). Altered microbiomes distinguish Alzheimer’s disease from amnestic mild cognitive impairment and health in a Chinese cohort. Brain Behav. Immun. 80, 633–643. 10.1016/j.bbi.2019.05.008 31063846

[B40] Liu S. Li E. Sun Z. Fu D. Duan G. Jiang M.. (2019). Altered gut microbiota and short chain fatty acids in Chinese children with autism spectrum disorder. Sci. Rep. 9 (1), 287. 10.1038/s41598-018-36430-z 30670726 PMC6342986

[B41] Louis P. Flint H. J. (2017). Formation of propionate and butyrate by the human colonic microbiota. Environ. Microbiol. 19 (1), 29–41. 10.1111/1462-2920.13589 27928878

[B42] Manderino L. Carroll I. Azcarate-Peril M. A. Rochette A. Heinberg L. Peat C.. (2017). Preliminary Evidence for an Association Between the Composition of the Gut Microbiome and Cognitive Function in Neurologically Healthy Older Adults. J. Int. Neuropsychol. Soc. 23 (8), 700–705. 10.1017/S1355617717000492 28641593 PMC6111127

[B43] Morris J. C. (1993). The Clinical Dementia Rating (CDR): current version and scoring rules. Neurology 43 (11), 2412–2414. 10.1212/wnl.43.11.2412-a 8232972

[B44] Morrison D. J. Preston T. (2016). Formation of short chain fatty acids by the gut microbiota and their impact on human metabolism. Gut Microbes 7 (3), 189–200. 10.1080/19490976.2015.1134082 26963409 PMC4939913

[B45] Nagpal R. Neth B. J. Wang S. Craft S. Yadav H. (2019). Modified Mediterranean-ketogenic diet modulates gut microbiome and short-chain fatty acids in association with Alzheimer’s disease markers in subjects with mild cognitive impairment. EBioMedicine 47, 529–542. 10.1016/j.ebiom.2019.08.032 31477562 PMC6796564

[B46] Nastasi C. Candela M. Bonefeld C. M. Geisler C. Hansen M. Krejsgaard T.. (2015). The effect of short-chain fatty acids on human monocyte-derived dendritic cells. Sci. Rep. 5 (1):16148. 10.1038/srep16148 26541096 PMC4635422

[B47] Parmentier-Decrucq E. Duhamel A. Ernst O. Fermont C. Louvet A. Vernier-Massouille G.. (2009). Effects of infliximab therapy on abdominal fat and metabolic profile in patients with Crohn’s disease. Inflammation Bowel Dis. 15 (10), 1476–1484. 10.1002/ibd.20931 19291781

[B48] Perez-Pardo P. Dodiya H. B. Engen P. A. Forsyth C. B. Huschens A. M. Shaikh M.. (2019). Role of TLR4 in the gut-brain axis in Parkinson’s disease: a translational study from men to mice. Gut 68 (5), 829–843. 10.1136/gutjnl-2018-316844 30554160

[B49] Qian Y. Yang X. Xu S. Wu C. Song Y. Qin N.. (2018). Alteration of the fecal microbiota in Chinese patients with Parkinson’s disease. Brain Behav. Immun. 70, 194–202. 10.1016/j.bbi.2018.02.016 29501802

[B50] Qiu X. Zhao X. Cui X. Mao X. Tang N. Jiao C.. (2020). Characterization of fungal and bacterial dysbiosis in young adult Chinese patients with Crohn’s disease. Ther. Adv. Gastroenterol 13, 320807230. 10.1177/1756284820971202 PMC7672770 33240394

[B51] Renbin L. Yawen Z. Luyi C. Yadong Q. Jiamin H. Mengjia H.. (2020). The effects of cigarettes and alcohol on intestinal microbiota in healthy men. J. Microbiol. 58 (11), 926–937. 10.1007/s12275-020-0006-7 32997305

[B52] Schaubeck M. Clavel T. Calasan J. Lagkouvardos I. Haange S. B. Jehmlich N.. (2016). Dysbiotic gut microbiota causes transmissible Crohn’s disease-like ileitis independent of failure in antimicrobial defence. Gut 65 (2), 225–237. 10.1136/gutjnl-2015-309333 25887379 PMC4752651

[B53] Singh H. Torralba M. G. Moncera K. J. DiLello L. Petrini J. Nelson K. E.. (2019). Gastro-intestinal and oral microbiome signatures associated with healthy aging. Geroscience 41 (6), 907–921. 10.1007/s11357-019-00098-8 31620923 PMC6925087

[B54] Sperling R. A. Aisen P. S. Beckett L. A. Bennett D. A. Craft S. Fagan A. M.. (2011). Toward defining the preclinical stages of Alzheimer’s disease: recommendations from the National Institute on Aging-Alzheimer’s Association workgroups on diagnostic guidelines for Alzheimer’s disease. Alzheimers Dement. 7 (3), 280–292. 10.1016/j.jalz.2011.03.003 21514248 PMC3220946

[B55] Stadlbauer V. Engertsberger L. Komarova I. Feldbacher N. Leber B. Pichler G.. (2020). Dysbiosis, gut barrier dysfunction and inflammation in dementia: a pilot study. BMC Geriatrics 20 (1), 248. 10.1186/s12877-020-01644-2 32690030 PMC7372911

[B56] Takeshita K. Mizuno S. Mikami Y. Sujino T. Saigusa K. Matsuoka K.. (2016). A Single Species of Clostridium Subcluster XIVa Decreased in Ulcerative Colitis Patients. Inflammatory Bowel Dis. 22 (12), 2802–2810. 10.1097/MIB.0000000000000972 27824645

[B57] Tamanai-Shacoori Z. Smida I. Bousarghin L. Loreal O. Meuric V. Fong S. B.. (2017). Roseburia spp.: a marker of health? Future Microbiol. 12, 157–170. 10.2217/fmb-2016-0130 28139139

[B58] Ticinesi A. Nouvenne A. Tana C. Prati B. Meschi T. (2019). “Gut Microbiota and Microbiota-Related Metabolites as Possible Biomarkers of Cognitive Aging (Cham: Springer International Publishing), 129–154. 10.1007/978-3-030-25650-0_8 31493226

[B59] Tran T. T. T. Corsini S. Kellingray L. Hegarty C. Le Gall G. Narbad A.. (2019). APOE genotype influences the gut microbiome structure and function in humans and mice: relevance for Alzheimer’s disease pathophysiology. FASEB J. 33 (7), 8221–8231. 10.1096/fj.201900071R 30958695 PMC6593891

[B60] Tremlett H. Bauer K. C. Appel-Cresswell S. Finlay B. B. Waubant E. (2017). The gut microbiome in human neurological disease: A review. Ann. Neurol. 81 (3), 369–382. 10.1002/ana.24901 28220542

[B61] Valech N. Tort-Merino A. Coll-Padrós N. Olives J. León M. Rami L.. (2018). Executive and Language Subjective Cognitive Decline Complaints Discriminate Preclinical Alzheimer’s Disease from Normal Aging. J. Alzheimer’s Dis. 61 (2), 689–703. 10.3233/JAD-170627 29254090

[B62] van Trijp M. P. H. Schutte S. Esser D. Wopereis S. Hoevenaars F. P. M. Hooiveld G. J. E. J.. (2020). Minor Changes in the Composition and Function of the Gut Microbiota During a 12-Week Whole Grain Wheat or Refined Wheat Intervention Correlate with Liver Fat in Overweight and Obese Adults. J. Nutr. 151 (3), 491–502. 10.1093/jn/nxaa312 PMC7948209 33188417

[B63] Vogt N. M. Kerby R. L. Dill-McFarland K. A. Harding S. J. Merluzzi A. P. Johnson S. C.. (2017). Gut microbiome alterations in Alzheimer’s disease. Sci. Rep. 7 (1), 13537. 10.1038/s41598-017-13601-y 29051531 PMC5648830

[B64] Vutukuri R. Brunkhorst R. Kestner R. I. Hansen L. Bouzas N. F. Pfeilschifter J.. (2018). Alteration of sphingolipid metabolism as a putative mechanism underlying LPS-induced BBB disruption. J. Neurochem. 144 (2), 172–185. 10.1111/jnc.14236 29023711

[B65] Wang Y. Sun L. Chen S. Guo S. Yue T. Hou Q.. (2019). The administration of Escherichia coli Nissle 1917 ameliorates irinotecan–induced intestinal barrier dysfunction and gut microbial dysbiosis in mice. Life Sci. 231, 116529. 10.1016/j.lfs.2019.06.004 31173781

[B66] Wang P. (2012). Tu1343 Butyrate Enhances Intestinal Epithelial Barrier Function via Up-Regulation of Tight Junction Protein Claudin-1 Transcription. Gastroenterol. (N. Y. N. Y. 1943) 142 (5), 807. 10.1016/S0016-5085(12)63137-0 22684624

[B67] Waters J. L. Ley R. E. (2019). The human gut bacteria Christensenellaceae are widespread, heritable, and associated with health. BMC Biol. 17 (1), 83. 10.1186/s12915-019-0699-4 31660948 PMC6819567

[B68] Wei H. Qing Y. Pan W. Zhao H. Li H. Cheng W.. (2013). Comparison of the efficiency of Banna miniature inbred pig somatic cell nuclear transfer among different donor cells. PloS One 8 (2), e57728. 10.1371/journal.pone.0057728 23469059 PMC3585185

[B69] Wei X. Tao J. Xiao S. Jiang S. Shang E. Zhu Z.. (2018). Xiexin Tang improves the symptom of type 2 diabetic rats by modulation of the gut microbiota. Sci. Rep. 8 (1), 3612–3685. 10.1038/s41598-018-22094-2 29487347 PMC5829262

[B70] Weis S. Schwiertz A. Unger M. M. Becker A. Fassbender K. Ratering S.. (2019). Effect of Parkinson’s disease and related medications on the composition of the fecal bacterial microbiota. NPJ Parkinsons Dis. 5, 28. 10.1038/s41531-019-0100-x 31815177 PMC6884491

[B71] Wu W. Lv L. Shi D. Ye J. Fang D. Guo F.. (2017). Protective Effect of Akkermansia muciniphila against Immune-Mediated Liver Injury in a Mouse Model. Front. Microbiol. 8:1804:1804. 10.3389/fmicb.2017.01804 29033903 PMC5626943

[B72] Zhang Y. Chen L. Hu M. Kim J. J. Lin R. Xu J.. (2020). Dietary type 2 resistant starch improves systemic inflammation and intestinal permeability by modulating microbiota and metabolites in aged mice on high-fat diet. Aging (Albany NY) 12 (10), 9173–9187. 10.18632/aging.103187 32452830 PMC7288951

[B73] Zhao Q. Guo Q. Li F. Zhou Y. Wang B. Hong Z.. (2013a). The Shape Trail Test: application of a new variant of the Trail making test. PloS One 8 (2), e57333. 10.1371/journal.pone.0057333 23437370 PMC3577727

[B74] Zhao Q. Guo Q. Hong Z. (2013b). Clustering and switching during a semantic verbal fluency test contribute to differential diagnosis of cognitive impairment. Neurosci. Bull. 29 (1), 75–82. 10.1007/s12264-013-1301-7 23322003 PMC5561862

[B75] Zhao Y. Cong L. Jaber V. Lukiw W. J. (2017). Microbiome-Derived Lipopolysaccharide Enriched in the Perinuclear Region of Alzheimer’s Disease Brain. Front. Immunol. 8, 1064. 10.3389/fimmu.2017.01064 28928740 PMC5591429

[B76] Zhao Y. Sharfman N. M. Jaber V. R. Lukiw W. J. (2019). Down-Regulation of Essential Synaptic Components by GI-Tract Microbiome-Derived Lipopolysaccharide (LPS) in LPS-Treated Human Neuronal-Glial (HNG) Cells in Primary Culture: Relevance to Alzheimer’s Disease (AD). Front. Cell Neurosci. 13, 314. 10.3389/fncel.2019.00314 31354434 PMC6635554

[B77] Zheng L. Kelly C. J. Battista K. D. Schaefer R. Lanis J. M. Alexeev E. E.. (2017). Microbial-Derived Butyrate Promotes Epithelial Barrier Function through IL-10 Receptor-Dependent Repression of Claudin-2. J. Immunol. 199 (8), 2976–2984. 10.4049/jimmunol.1700105 28893958 PMC5636678

[B78] Zhu H. Liu Y. Li S. Jin Y. Zhao L. Zhao F.. (2018). Altered gut microbiota after traumatic splenectomy is associated with endotoxemia. Emerg. Microbes Infect. 7 (1), 197. 10.1038/s41426-018-0202-2 30498207 PMC6265257

[B79] Zhuang Z. Shen L. Li W. Fu X. Zeng F. Gui L.. (2018). Gut Microbiota is Altered in Patients with Alzheimer’s Disease. J. Alzheimer’s Dis. 63 (4), 1337–1346. 10.3233/JAD-180176 29758946

