# Supplementary figures and images for: Preoperative Microbiomes and Intestinal Barrier Function Can Differentiate Prodromal Alzheimer’s Disease From Normal Neurocognition in Elderly Patients Scheduled to Undergo Orthopedic Surgery
Source: Front Cell Infect Microbiol. 2021 Mar 29;11:592842. doi: 10.3389/fcimb.2021.592842 (PMC8044800; doi:10.3389/fcimb.2021.592842)

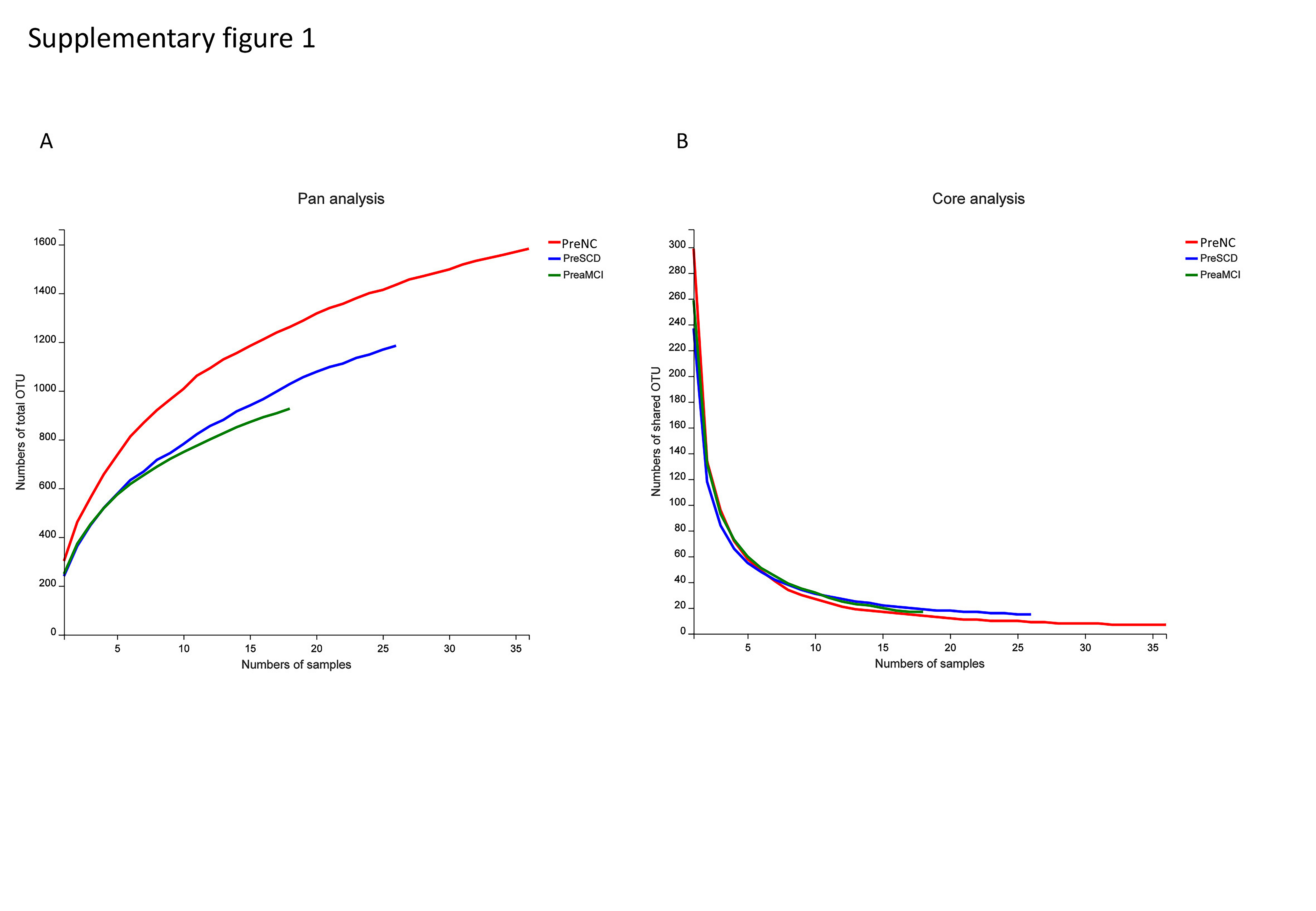

Supplement: Supplementary Figure 1 — (A, B) OTU analysis of three groups. The horizontal axis represents the number of samples observed, and the vertical axis represents the total/core species of all samples under a grouping category. Pan OTU is the sum of the OTU contained in all samples, which is used to observe the increase of the total number of OTU as the number of samples increases. Core OTU refers to the number of common OTU in all samples, which is used to observe the decrease of the number of common OTU as the number of samples increases. Red, blue, and green curves represent the NC, SCD, and aMCI groups’ OTU analysis. [file Image_1.jpg]
