# Supplementary material for: Preoperative Microbiomes and Intestinal Barrier Function Can Differentiate Prodromal Alzheimer’s Disease From Normal Neurocognition in Elderly Patients Scheduled to Undergo Orthopedic Surgery
Source: Front Cell Infect Microbiol. 2021 Mar 29;11:592842. doi: 10.3389/fcimb.2021.592842 (PMC8044800; doi:10.3389/fcimb.2021.592842)
Supplement: Supplementary file 2 [file Table_1.docx]

**[Supplementary](C:/Users/duanmei/AppData/Local/youdao/dict/Application/7.5.2.0/resultui/dict/?keyword=supplementary) Table 1 | Statistical data of sample sequence information**

| Sample | Group | Seq_num | Base_num | Mean_length | Min_length | Max_length |
| --- | --- | --- | --- | --- | --- | --- |
| G1 | NC | 54633 | 24081715 | 440.7906394 | 283 | 451 |
| G2 | SCD | 61653 | 27068378 | 439.0439719 | 358 | 462 |
| G3 | NC | 66250 | 28771156 | 434.2816 | 337 | 532 |
| G4 | SCD | 47546 | 20929767 | 440.2003744 | 312 | 452 |
| G5 | aMCI | 57478 | 24966301 | 434.3627301 | 282 | 504 |
| G6 | NC | 48497 | 20964307 | 432.2804916 | 310 | 452 |
| G7 | aMCI | 68381 | 29882025 | 436.9930975 | 312 | 452 |
| G8 | SCD | 73924 | 32076329 | 433.9095422 | 273 | 455 |
| G9 | NC | 57066 | 24752097 | 433.7450846 | 276 | 451 |
| G10 | NC | 58841 | 26159666 | 444.5822811 | 283 | 474 |
| G11 | SCD | 53861 | 23715720 | 440.3133993 | 283 | 451 |
| G12 | SCD | 73490 | 32234795 | 438.6283168 | 283 | 460 |
| G13 | NC | 49755 | 21793690 | 438.0200985 | 412 | 453 |
| G14 | SCD | 51637 | 22824983 | 442.027674 | 366 | 454 |
| G15 | SCD | 58991 | 26094100 | 442.340357 | 421 | 462 |
| G16 | NC | 49531 | 21697490 | 438.0587915 | 333 | 461 |
| G17 | SCD | 61452 | 26712119 | 434.6826629 | 386 | 452 |
| G18 | SCD | 59734 | 26026969 | 435.7144842 | 344 | 452 |
| G19 | NC | 53898 | 23891959 | 443.2809937 | 366 | 452 |
| G20 | NC | 58653 | 25171445 | 429.1586961 | 400 | 451 |
| G21 | NC | 53267 | 23429136 | 439.8433552 | 414 | 473 |
| G22 | NC | 42160 | 18722673 | 444.0861717 | 379 | 453 |
| S1 | NC | 94641 | 40960864 | 432.8025274 | 357 | 532 |
| S2 | aMCI | 70374 | 30466521 | 432.9229687 | 321 | 473 |
| S3 | aMCI | 49472 | 21540102 | 435.3998625 | 358 | 461 |
| S4 | NC | 96327 | 41859665 | 434.557964 | 225 | 460 |
| S5 | NC | 103701 | 44484795 | 428.9717071 | 390 | 452 |
| S6 | NC | 99053 | 43109784 | 435.2193674 | 358 | 470 |
| S7 | aMCI | 60686 | 26029186 | 428.915829 | 305 | 452 |
| S8 | NC | 50814 | 22051747 | 433.9699099 | 358 | 452 |
| S9 | NC | 44528 | 19503496 | 438.0052102 | 379 | 453 |
| S10 | aMCI | 43587 | 18997021 | 435.8414436 | 417 | 451 |
| S11 | NC | 46776 | 20250938 | 432.9343681 | 328 | 451 |
| S12 | aMCI | 42571 | 18475923 | 434.0025604 | 281 | 467 |
| S13 | aMCI | 50918 | 21874833 | 429.6090381 | 394 | 451 |
| S14 | SCD | 43694 | 19123696 | 437.6732732 | 366 | 452 |
| S15 | SCD | 49632 | 21494675 | 433.080976 | 358 | 452 |
| S16 | NC | 60412 | 26097406 | 431.9904324 | 275 | 453 |
| S17 | NC | 51633 | 22491750 | 435.6080414 | 312 | 455 |
| S18 | NC | 50554 | 22006594 | 435.30866 | 397 | 452 |
| S19 | aMCI | 71689 | 30895858 | 430.9706929 | 358 | 476 |
| S20 | SCD | 36854 | 16026960 | 434.8770825 | 407 | 452 |
| S21 | NC | 59699 | 26140156 | 437.8658939 | 366 | 452 |
| S22 | aMCI | 56878 | 24616756 | 432.7992545 | 385 | 452 |
| S23 | NC | 51596 | 22320712 | 432.6054733 | 383 | 464 |
| S24 | NC | 68327 | 29909516 | 437.7408052 | 273 | 473 |
| S25 | NC | 53221 | 23618532 | 443.7821912 | 273 | 451 |
| S26 | SCD | 59786 | 26046244 | 435.6579132 | 360 | 453 |
| S27 | NC | 47684 | 20487113 | 429.6433395 | 416 | 451 |
| S28 | aMCI | 51870 | 22614603 | 435.986177 | 291 | 453 |
| S29 | aMCI | 45468 | 19854046 | 436.6597607 | 415 | 452 |
| S30 | SCD | 60353 | 26352547 | 436.6402167 | 346 | 451 |
| S31 | SCD | 57277 | 24783931 | 432.7030222 | 338 | 451 |
| S32 | SCD | 51267 | 22423555 | 437.3876958 | 331 | 452 |
| S33 | SCD | 52797 | 23001403 | 435.6573858 | 344 | 451 |
| S34 | NC | 51802 | 22606472 | 436.4015289 | 273 | 452 |
| S35 | NC | 49370 | 21755721 | 440.666822 | 298 | 457 |
| S36 | NC | 49086 | 21390579 | 435.7775944 | 307 | 452 |
| S37 | SCD | 56245 | 24286434 | 431.7972086 | 274 | 452 |
| S38 | aMCI | 41486 | 17957316 | 432.8524321 | 283 | 452 |
| S39 | aMCI | 46321 | 20314951 | 438.5689212 | 283 | 451 |
| S40 | NC | 46774 | 20445210 | 437.1062984 | 274 | 453 |
| S41 | SCD | 45461 | 19585139 | 430.8118827 | 349 | 469 |
| S42 | NC | 44100 | 19090835 | 432.8987528 | 312 | 451 |
| S43 | aMCI | 52456 | 23074163 | 439.8765251 | 307 | 452 |
| S44 | SCD | 45278 | 19634679 | 433.6472238 | 277 | 451 |
| S45 | SCD | 47657 | 20842999 | 437.3544075 | 286 | 452 |
| S46 | NC | 52989 | 23409838 | 441.7867482 | 274 | 512 |
| S47 | aMCI | 41778 | 18362063 | 439.5151276 | 389 | 451 |
| S48 | NC | 53256 | 23338503 | 438.2323682 | 359 | 454 |
| S49 | aMCI | 46153 | 20078945 | 435.0517843 | 356 | 495 |
| S50 | NC | 52875 | 23136347 | 437.5668463 | 273 | 452 |
| S51 | SCD | 68055 | 29588802 | 434.7777827 | 357 | 495 |
| S52 | SCD | 57430 | 24980007 | 434.9644263 | 346 | 452 |
| S53 | SCD | 53214 | 23417236 | 440.0578043 | 283 | 451 |
| S54 | NC | 69032 | 29595723 | 428.7246929 | 306 | 475 |
| S55 | aMCI | 56759 | 25134598 | 442.8301767 | 283 | 489 |
| S56 | NC | 60619 | 26472722 | 436.7066761 | 282 | 477 |
| S57 | SCD | 57366 | 25438360 | 443.4396681 | 366 | 452 |
| S58 | SCD | 56546 | 25008597 | 442.2699572 | 366 | 452 |
| Total | 80 | 4496945 | 1960823987 | 436.2227439 | / | / |

Note: Seq_num: Number of sample sequence reads; Base_num: Number of sample base reads; Mean_length: The mean length of sample sequence; Min_length: The min length of sample sequence; Max_length: The max length of sample sequence.
