# Supplementary material for: Preoperative Microbiomes and Intestinal Barrier Function Can Differentiate Prodromal Alzheimer’s Disease From Normal Neurocognition in Elderly Patients Scheduled to Undergo Orthopedic Surgery
Source: Front Cell Infect Microbiol. 2021 Mar 29;11:592842. doi: 10.3389/fcimb.2021.592842 (PMC8044800; doi:10.3389/fcimb.2021.592842)
Supplement: Supplementary file 3 [file Table_2.docx]

| KO functional categories | | NC mean (SD) | SCD mean (SD) | aMCI mean (SD) | NC *vs* SCD | NC *vs* aMCI | SCD *vs* aMCI |
| --- | --- | --- | --- | --- | --- | --- | --- |
| Level1 | Level2 | *10^5^ Abundance | | | P value | P value | P value |
| Cellular Processes | Transport and Catabolism | 1.307(0.369) | 1.223(0.292) | 1.114(0.267) | — | — | — |
| Environmental Information Processing | Membrane Transport | 56.910(24.490) | 45.923(12.545) | 49.685(18.850) | — | — | — |
| Environmental Information Processing | Signal Transduction | 7.647(3.939) | 5.981(1.580) | 6.160(1.852) | — | — | — |
| Environmental Information Processing | Signaling Molecules and Interaction | 0.706(0.213) | 0.636(0.154) | 0.586(0.139) | — | 0.007 | — |
| Genetic Information Processing | Replication and Repair | 40.481(15.476) | 33.710(6.632) | 33.851(10.233) | 0.04 | 0.034 | — |
| Genetic Information Processing | Transcription | 13.677(5.840) | 11.057(2.548) | 11.590(3.819) | — | — | — |
| Genetic Information Processing | Translation | 25.583(10.533) | 21.037(4.290) | 21.371(6.841) | — | — | — |
| Metabolism | Amino Acid Metabolism | 44.847(17.819) | 36.777(8.139) | 37.755(11.855) | 0.029 | — | — |
| Metabolism | Carbohydrate Metabolism | 49.018(18.254) | 40.653(9.005) | 42.034(12.472) | — | — | — |
| Metabolism | Energy Metabolism | 26.314(9.719) | 22.098(4.569) | 22.284(6.957) | 0.041 | 0.035 | — |
| Metabolism | Glycan Biosynthesis and Metabolism | 10.246(2.852) | 9.291(1.713) | 8.532(1.866) | — | 0.004 | 0.045 |
| Metabolism | Lipid Metabolism | 13.426(5.275) | 10.922(2.432) | 11.135(3.351) | 0.034 | 0.015 | — |
| Metabolism | Metabolism of Cofactors and Vitamins | 20.374(7.838) | 17.028(3.398) | 17.035(5.269) | 0.044 | 0.034 | — |
| Metabolism | Xenobiotics Biodegradation and Metabolism | 7.036(2.233) | 5.852(1.409) | 6.023(1.877) | 0.031 | 0.009 | — |
| Organismal Systems | Endocrine System | 1.502(0.562) | 1.270(0.277) | 1.246(0.328) | — | — | — |
| Organismal Systems | Environmental Adaptation | 0.841(0.538) | 0.640(0.181) | 0.649(0.232) | — | — | — |
| Organismal Systems | Immune System | 0.425(0.175) | 0.350(0.076) | 0.351(0.106) | 0.043 | 0.035 | — |
| Organismal Systems | Nervous System | 0.508(0.189) | 0.430(0.111) | 0.428(0.148) | — | 0.029 | — |

**Supplementary Table 2 | Predicted KEGG functional pathways differences at level 2 inferred from 16S rRNA gene sequences using PICRUSt.**

Note: Data are given as mean (SD). We applied non-parametric rank sum test in PICRUSt analysis between different cognitive groups. “—” means that there were not significant differences among three groups. Abbreviations: NC, normal neurocognition; SCD, subjective cognitive decline; aMCI, amnestic mild cognitive impairment; KEGG, Kyoto Encyclopedia of Genes and Genomes; PICRUSt, Phylogenetic Investigation of Communities by Reconstruction of Unobserved States; KO, KEGG Ortholog; SD: standard deviation.
